# Prenatal Prescription Opioid Analgesic Exposure and Academic Performance in Third Grade Children: A Population‐Based Cohort Study

**DOI:** 10.1111/1471-0528.70221

**Published:** 2026-03-15

**Authors:** Bianca Varney, Helga Zoega, Malcolm B. Gillies, Claudia Bruno, Alys Havard, Antonia Shand, Natasha Nassar, Jonathan Brett

**Affiliations:** ^1^ Medicines Intelligence Research Program, School of Population Health, Faculty of Medicine and Health University of New South Wales Sydney Australia; ^2^ National Drug and Alcohol Research Centre, Faculty of Medicine and Health, UNSW Sydney Sydney Australia; ^3^ Centre of Public Health Sciences, Faculty of Medicine University of Iceland Reykjavík Iceland; ^4^ Leeder Centre for Health Policy, Economics and Data, Faculty of Medicine and Health, University of Sydney Sydney Australia; ^5^ Child Population and Translational Health Research, Children's Hospital at Westmead Clinical School, Faculty of Medicine and Health University of Sydney Sydney Australia; ^6^ Charles Perkins Centre University of Sydney Sydney Australia; ^7^ Department of Maternal Fetal Medicine, Royal Hospital for Women, Randwick Sydney Australia; ^8^ Children's Hospital at Westmead Clinical School, Faculty of Medicine and Health Sydney Australia; ^9^ St. Vincent's Clinical School, Faculty of Medicine and Health University of New South Wales Sydney Australia

## Abstract

**Objective:**

To evaluate the association between prenatal exposure to opioids analgesics and academic performance in third grade children.

**Design:**

Population‐based cohort study using linked data from New South Wales birth records, medicine dispensing data, and national standardised test results.

**Setting:**

New South Wales, Australia (2003–2011).

**Population:**

Liveborn children of concessional women, excluding those whose mothers lived interstate, were overseas visitors, or had records of opioid dependence.

**Methods:**

Exposure was defined as ≥ 1 opioid dispensed during pregnancy stratified by pregnancy timing, dose, and type of monotherapy. Reading and numeracy z‐scores were compared between children with and without prenatal opioid exposure using linear mixed‐effects models incorporating propensity score weights and accounting for maternal and school clustering.

**Main Outcome Measures:**

Standardised national reading and numeracy Z‐scores for third‐grade children.

**Results:**

Among 85,478 eligible children, 70,882 (82.9%) had test scores, with 7,664 (10.2%) prenatally exposed to opioids analgesics, mainly codeine. Prenatal e.xposure to opioid analgesics was associated with a slight decrease in standardised reading and numeracy test scores (adjusted mean difference [aβ] −0.05, 95% CI −0.06 to −0.03), below the conventional threshold for a small effect (Cohen's *d* = 0.2). Codeine and oxycodone results were similar to those for any opioid exposure, while a larger difference was observed for tramadol (reading aβ −0.25, 95% CI −0.31 to −0.18; numeracy aβ −0.22, 95% CI −0.29 to −0.16).

**Conclusions:**

Prenatal exposure to codeine and oxycodone do not have meaningful impacts on third‐grade academic performance. In contrast, tramadol was associated with lower reading and numeracy scores, though whether this reflects a true effect or is attributable to unmeasured confounding remains unclear and warrants further investigation.

**Abbreviations**
aβ: adjusted beta
CI: confidence interval
ED: emergency department
ELC: early life course
GP: general practitioner
HIV: human immunodeficiency virus
IPCW: inverse probability of censoring weighting
IQR: interquartile range
LMP: last menstrual period
NAPLAN: national assessment program—literacy and numeracy
NMS: National Minimum Standard
NSW: New South Wales
OAT: opioid agonist therapy
OD: opioid dependence
OME: oral morphine equivalent
OTC: over the counter
OUD: opioid use disorder
PBS: pharmaceutical benefits scheme
RR: relative risk
SD: standard deviation
STROBE: strengthening the reporting of observational studies in epidemiology

## 1 Introduction

Pain is a commonly reported symptom during pregnancy [1], such as pain from lumbopelvic [2] or pelvic girdle conditions [3]. Due to limited alternatives, opioid analgesics are often used to treat pain during pregnancy, with global prevalence ranging from 4 to 191 per 1000 pregnancies [4]. However, opioids freely cross the placenta resulting in fetal exposure. Animal studies have demonstrated that opioids inhibit neuronal development in the central nervous system (CNS) by altering growth factors and signalling molecules involved in neurogenesis and apoptosis [5, 6, 7]. Human studies of infants with neonatal abstinence syndrome (NAS) following prenatal opioid exposure have reported reduced plasma concentrations of proteins critical for neuronal survival, growth, and plasticity, key processes underlying learning and memory [8, 9]. Therefore, it is biologically plausible that prenatal opioid exposure could disrupt fetal CNS development, contributing to later difficulties with academic skills such as reading and numeracy.

Three studies from the Norwegian Mother, Father, and Child Cohort (MoBa) have examined prenatal opioid analgesic exposure and childhood educational outcomes, finding no clinically meaningful increased risk of impaired language development or academic performance [10, 11, 12]. However, none of the studies explored the effects of cumulative dose nor individual opioid monotherapies, which may have different risks. This highlights the need for further research to replicate these findings in other cohorts and expand our understanding of the long‐term safety of opioid analgesics during pregnancy.

Our study investigated the association between prenatal exposure to opioid analgesics and academic performance on reading and numeracy tests in third grade children. We also aimed to examine whether these associations varied by opioid type, timing of exposure, or cumulative dose.

## 2 Methods

### 2.1 Study Setting, Study Design and Data Sources

We conducted a population‐based cohort study of all liveborn children (≥ 400 g or ≥ 20 weeks gestation) in New South Wales (NSW), Australia, using the Early Life Course (ELC) data [13]. The ELC dataset comprises linked administrative records including perinatal data, school performance records, social security information, hospital admissions, emergency department (ED) presentations, records of opioid agonist treatment (OAT) for opioid dependence (OD), mortality data, and dispensing records for medicines subsidised through the Pharmaceutical Benefits Scheme (PBS). See Appendix S1 for a more detailed description of the data sources. We report our study in accordance with Strengthening the Reporting of Observational Studies in Epidemiology (STROBE) guidelines (Table S9).

### 2.2 Study Population

We identified all liveborn children in NSW whose mothers had an estimated last menstrual period (LMP; date of childbirth—gestational age at delivery [weeks] × 7) between January 1, 2003, and March 26, 2011. The study's end date was selected to ensure that most children reached the required age of (8–9 years) for participation in third grade national standardised testing and to avoid overrepresenting pregnancies shorter than 40 weeks' gestation. Our cohort was restricted to children born to women with continuous concessional beneficiary status from 365 days before their LMP until childbirth. We restricted to this population because before July 2012, the PBS database only recorded medicines dispensed that cost more than the co‐payment threshold. The Australian government subsidises PBS‐listed medicines when their prices are above these thresholds. Concessional beneficiaries, including social security recipients, low‐income earners, individuals with disabilities, and caregivers, pay a reduced co‐payment ($7.70 in 2024) compared to general beneficiaries ($31.60). Since all medicines cost more than the concessional co‐payment threshold, complete dispensing data were only available for concessional beneficiaries.

We excluded children with missing or implausible gestational age (*n* = 8), stillbirths (*n* = 784), those whose mothers were interstate residents or overseas visitors (*n* = 951), and those whose mothers had an OD diagnosis or received OAT from 12 months before LMP until childbirth (*n* = 1091) (Table S1—exclusion criteria codes). Figure S1—illustrates the study design.

### 2.3 Exposure

We included all PBS‐subsidised opioid analgesics available during the study period (Table S2). Pharmaceutical dispensing records provide an objective, prospectively collected measure of medicine exposure that is routinely used in pharmacoepidemiological research [14, 15]. During the study period, methadone and buprenorphine were PBS‐subsidised only for the indication of pain. Children were classified as prenatally exposed if their mother was dispensed ≥ 1 opioid analgesic during pregnancy (LMP until childbirth), and unexposed if no opioid was dispensed from 90 days before LMP through childbirth. As synaptogenesis is known to peak during late pregnancy [16], we examined exposure timing across distinct gestational periods: early pregnancy only (LMP through until gestational week 20 with no late pregnancy exposure), late pregnancy only (gestational week 20 until childbirth with no early pregnancy exposure), and both early and late pregnancy exposure.

To examine effects of the most common individual opioids, we stratified exposure into monotherapy groups for codeine (including combinations with paracetamol or aspirin), oxycodone (including oxycodone‐naloxone), and tramadol, where women received only that specific opioid type during pregnancy. We also evaluated cumulative opioid dose by calculating total oral morphine equivalents (OME), dichotomised into low (< 60 mg) OME and higher (≥ 60 mg) OME. We calculated total OME by multiplying each dispensed opioid's strength (mg), quantity, and OME conversion factor [17], then summing the OME for all opioids dispensed during pregnancy.

### 2.4 Academic Performance

Academic performance was assessed through third grade reading and numeracy scores from the National Assessment Program—Literacy and Numeracy (NAPLAN), an annual standardised assessment administered nationwide by each state and territory (in our case NSW) since 2008 that evaluates students' proficiency in reading, writing, language conventions, and numeracy. We focused on reading and numeracy scores as these domains have demonstrated the highest stability across multiple testing periods [18]. We calculated z‐scores using the mean and standard deviation (SD) for the first available third‐grade test results for all children in our cohort who completed each subject in a given assessment year.

As a secondary outcome, we examined whether third‐grade students scored below the National Minimum Standard (NMS), representing those who lack the fundamental skills needed for academic progression and are typically targeted for educational interventions [19].

### 2.5 Covariates

Covariates included maternal, parental, and child‐related factors, with the corresponding codes, data sources, and ascertainment windows detailed in Table S3. Maternal factors included sociodemographics, comorbidities, medicine use, and pre‐pregnancy healthcare utilisation as a marker of a woman's overall comorbidity burden. Parental characteristics included highest education level and occupation of first and second parent (if available). Child‐related factors comprised birth year, sex, year of testing, and primary language spoken at home. A directed acyclic graph informed the identification of confounders (Figure S2). Missing data were addressed by assigning a “missing” category (for child's sex, language spoken at home, and parental education and occupation), and by imputing values using the mode (partner status, parity, previous caesarean delivery) or the median (maternal age, area‐based socioeconomic disadvantage, and remoteness) (Table S3).

### 2.6 Follow‐Up and Missing Test Scores

Follow‐up commenced at childbirth and continued until the earliest occurrence of an outcome, death, or the administrative end of the study (31st December 2019). Children who died before age nine were classified as deceased; those younger than 9 years old at the last available test date (31st May 2019) were classified as too young for testing. Missing test scores occurred in two scenarios: non‐participation in NAPLAN tests (absences, withdrawals, or exemptions for intellectual disability, significant co‐existing conditions, or limited English proficiency) and no NAPLAN records (classified as lost to follow‐up).

### 2.7 Statistical Analysis

Among children with prenatal exposure to opioid analgesics, we characterised exposure profiles by examining the proportion with 1, 2, or ≥ 3 dispensings and reporting median and interquartile (IQR) range of total OME, stratified by opioid type. We compared covariate characteristics between: (1) children with prenatal opioid exposure (any or specific types) and unexposed children, (2) children with test scores and those lost to follow‐up to evaluate attrition bias, and (3) children included in our cohort and those born to women who did not meet the continuous concessional beneficiary definition (i.e., representing the remainder of the NSW population) to evaluate generalisability. For these comparisons, an absolute standardised mean difference > 0.1 indicated meaningful group differences.

### 2.8 Primary Analysis

We assessed the total effect of prenatal opioid exposure on either reading or numeracy z‐scores using mixed‐effects linear regression models with random intercepts for school and maternal clustering. Models yielded beta (*β*) coefficients with 95% confidence intervals calculated using Satterthwaite's approximation [20]. To control for confounding and ensure balance across maternal, paternal and child‐related characteristics, we applied inverse probability weighting incorporating all covariates listed in the covariate section, including those related to the outcome regardless of their association with the exposure [21]. Propensity scores were calculated using logistic regression, with maternal age modelled as a natural cubic spline with 4 degrees of freedom, selected based on the Akaike Information Criterion. Beta coefficients > 0.2 were considered indicative of meaningful academic differences [22].

For our secondary outcome, we calculated the relative risk (RR) of performing below the NMS using Poisson regression with a log‐link function. Models used generalized estimating equations with an exchangeable correlation structure to account for school clustering, and adjusted analyses incorporated propensity score weights. All analyses were restricted to children with available test scores.

### 2.9 Sensitivity Analysis

We conducted several sensitivity analyses to explore the robustness of our findings. To test the robustness of the findings to potential exposure misclassification, we redefined exposure as ≥ 2 dispensing records during pregnancy, as multiple dispensings are more likely to reflect actual medicine use and are commonly used to strengthen confidence in exposure classification [23]. To examine potential unmeasured confounding, we redefined the unexposed group as children whose mothers discontinued opioid use before pregnancy (i.e., ≥ 1 opioid dispensing between 365 and 90 days prior to the LMP, with no dispensing from 90 days before LMP through the end of pregnancy) and conducted a sibling analysis restricted to exposure‐discordant siblings born to the same mother. To assess generalisability, we recalculated z‐scores for an expanded cohort including children born to both concessional and children born to women not meeting the continuous concessional definition. Finally, we assessed potential selection bias from restricting analyses to children with test scores using two approaches. Exempt students were either assigned z‐scores 2 SD below their exposure group mean or classified as below the NMS, allowing us to test whether the main findings were robust to different approaches for handling exclusions and potential differential exclusion by exposure group. Second, we applied inverse probability of censoring weights (IPCW) to account for all missing test data, including non‐participants (exempt, absent, or withdrawn) and those lost to follow‐up (methods in Appendix S2).

### 2.10 Post Hoc Analyses

We compared academic outcomes in children exposed to low versus higher OME during pregnancy to assess the dose–response relationship. For the potential safety signal associated with tramadol, we calculated the *E*‐value to estimate the strength needed for an unmeasured confounder to explain observed associations [24]. Finally, recognizing that prenatal opioid exposure may increase the risk of adverse birth outcomes [25, 26, 27, 28], which are risk factors for poor childhood development and academic performance [29, 30], we examined the potential role of these outcomes in the association between prenatal opioid exposure and academic achievement. We compared both crude and standardized proportions of adverse birth outcomes across exposure groups, with all estimates accounting for maternal clustering and standardized proportions incorporating propensity score weights based on all covariates except child sex and test year, as these are not confounders in the exposure‐outcome pathway (Table S4).

### 2.11 Ethics Approvals

The study received approval from the Australian Institute of Health and Welfare Human Research Ethics Committee (EO2020‐2‐1130) and the New South Wales Population and Health Services Research Ethics Committee (2019/ETH11830).

## 3 Results

**FIGURE 1:**
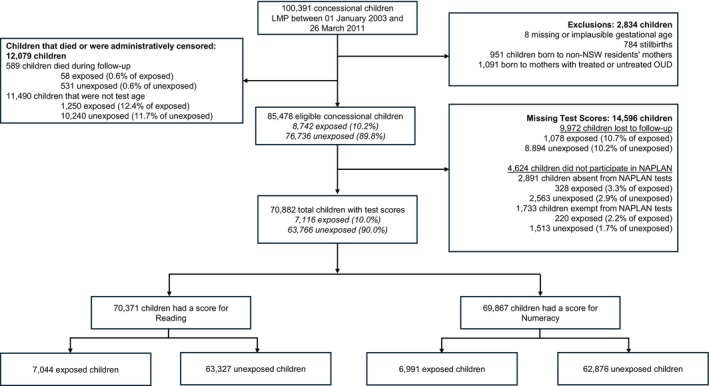
Study cohort flowchart. LMP, last menstrual period; NSW, New South Wales; NAPLAN, National Assessment Program—Literacy and Numeracy; OUD, opioid use disorder; SD, standard deviation.

There were 85 478 children born to concessional beneficiaries (64 812 unique mothers; 84 242 pregnancies) who were eligible to be included in the study cohort (Figure 1), of whom 70 882 (82.9%) children had test scores. There was no differential pattern of missing data between exposure groups among NAPLAN non‐participants and those lost to follow‐up (Figure 1). Furthermore, children lost to follow‐up had similar characteristics to those with test scores (Table S5).

Among children who had test scores, 7116 (10.0%) were prenatally exposed to analgesic opioids; 3084 (4.4%) in early pregnancy, 3083 (4.3%) in late pregnancy, and 949 (1.3%) in both early and late pregnancy. Codeine was the most common monotherapy (89.6% of exposures), followed by tramadol (3.5%) and oxycodone (2.3%). Most children were exposed to a single opioid dispensing during pregnancy (72.0%), with 14.8% exposed to two and 13.2% to three or more (Figure S3). Children prenatally exposed to tramadol had the highest median total OME (400 mg, IQR: 200–600), followed by oxycodone (150 mg, IQR: 150–300) and codeine (60 mg, IQR: 60–60) (Figure S4). Mothers of children who were prenatally exposed to opioids were more likely to smoke during pregnancy, have mental health conditions, use both opioid (pre‐pregnancy) and non‐opioid analgesics, and have higher pre‐pregnancy healthcare utilisation compared to mothers of children not exposed (Table 1, Table S6). After propensity score weighting, all covariates were balanced (Figure S5).

**TABLE 1:** Maternal and child characteristics by exposure to opioid analgesics during pregnancy, for children of concessional beneficiaries between January 01, 2003, to March 26, 2011.

Characteristics	Study cohort		Absolute standardised differences
	Unexposed to opioids (*n* = 63 776) no. (%)	Exposed to opioids (*n* = 7116) no. (%)	
*Child factors*			
Child's sex			
Male	31 716 (49.7)	3534 (49.7)	0.00
Female	32 042 (50.2)	^a^	0.00
Indeterminate/unknown	8 (0.0)	≤ 5	0.00
Child's year of birth			
2003	1502 (2.4)	178 (2.5)	0.01
2004	6068 (9.5)	638 (9.0)	0.02
2005	7163 (11.2)	852 (12.0)	0.02
2006	7815 (12.3)	843 (11.8)	0.01
2007	8461 (13.3)	938 (13.2)	0.00
2008	8989 (14.1)	1045 (14.7)	0.02
2009	9535 (15.0)	1039 (14.6)	0.01
2010	10 734 (16.8)	1184 (16.6)	0.01
2011	3499 (5.5)	399 (5.6)	0.01
Test year			
2011	≤ 5	0 (0.0)	≤ 0.01
2012	^a^	397 (5.3)	≤ 0.01
2013	6447 (10.1)	688 (9.7)	0.02
2014	7305 (11.5)	846 (11.9)	0.01
2015	8040 (12.6)	874 (12.3)	0.01
2016	8620 (13.5)	937 (13.2)	0.01
2017	9111 (14.3)	1065 (15.0)	0.02
2018	9778 (15.3)	1073 (15.1)	0.01
2019	11 099 (17.4)	1236 (17.4)	0.00
Language spoken at home			
English	45 879 (71.9)	5229 (73.5)	0.03
Non‐English language	17 005 (26.7)	1791 (25.2)	0.03
Missing	882 (1.4)	96 (1.3)	0.00
*Maternal factors*			
Maternal age at child's birth (years)	29.5 (5.9)	29.1 (5.8)	0.06
Mean (SD)			
< 25	14 766 (23.2)	1741 (24.5)	0.03
25 to 34	35 226 (55.2)	3949 (55.5)	0.01
35+	13 774 (21.6)	1426 (20.0)	0.04
Born in a Western Country	13 666 (21.4)	1388 (19.5)	0.05
Does not have a partner	21 910 (34.4)	3060 (43.0)	0.18
Social security benefits received^b^	14 405 (22.6)	1703 (23.9)	0.03
Parity			
First born child	4777 (7.5)	549 (7.7)	0.01
Second born child	22 090 (34.6)	2300 (32.3)	0.05
Third born child	18 458 (28.9)	1931 (27.1)	0.04
Fourth born child or more	18 441 (28.9)	2336 (32.8)	0.09
Previous caesarean delivery	29.1 (5.8)	1212 (17.0)	0.02
Smoking during pregnancy	4579 (7.2)	1440 (20.2)	0.39
Quintiles of area‐based socioeconomic disadvantage (IRSD)			
Most disadvantaged	28 141 (44.1)	3399 (47.8)	0.07
2	15 009 (23.5)	1739 (24.4)	0.02
3	11 760 (18.4)	1269 (17.8)	0.02
4	4948 (7.8)	440 (6.2)	0.06
Least disadvantaged	3908 (6.1)	269 (3.8)	0.11
Area‐based remoteness (ARIA)			
Major Cities of Australia	43 097 (67.6)	4888 (68.7)	0.02
Inner Regional Australia	14 479 (22.7)	1577 (22.2)	0.01
Outer Regional Australia	5571 (8.7)	598 (8.4)	0.01
Remote and very remote Australia	619 (1.0)	53 (0.7)	0.03
Multifetal gestation	1796 (2.8)	243 (3.4)	0.03
Parental^c^ highest educational attainment			
Bachelor's degree or above	7772 (12.2)	536 (7.5)	0.16
Certificate or diploma	30 929 (48.5)	3383 (47.5)	0.02
Year 12 or equivalent	6194 (9.7)	695 (9.8)	0.00
<Year 12	15 954 (25.0)	2166 (30.4)	0.12
Missing	2917 (4.6)	336 (4.7)	0.01
Parent occupation^d^			
Group 1	4004 (6.3)	299 (4.2)	0.09
Group 2	7966 (12.5)	604 (8.5)	0.13
Group 3	13 292 (20.8)	1363 (19.2)	0.04
Group 4	16 172 (25.4)	1861 (26.2)	0.02
No paid work in the previous 12 months	16 269 (25.5)	2236 (31.4)	0.13
Missing	6063 (9.5)	753 (10.6)	0.04
*Maternal conditions (year prior to LMP until the day before childbirth)*			
Anaemia and coagulation	989 (1.6)	227 (3.2)	0.11
Cardiovascular disease	1345 (2.1)	354 (5.0)	0.16
Cancer diagnosis or treatment	≤ 5	≤ 5	≤ 0.01
Chronic liver disease	24 (0.0)	≤ 5	≤ 0.01
Chronic renal disease	33 (0.1)	15 (0.2)	0.04
Drug and alcohol disorder	615 (1.0)	155 (2.2)	0.10
Epilepsy	64 (0.1)	12 (0.2)	0.02
Pre‐existing hypertension	301 (0.5)	41 (0.6)	0.01
Pre‐existing diabetes	286 (0.4)	40 (0.6)	0.02
Mental health	8859 (13.9)	2064 (29.0)	0.38
Severe mental health	775 (1.2)	189 (2.7)	0.11
Obesity	124 (0.2)	23 (0.3)	0.03
Respiratory disease	7 (0.0)	≤ 5	0.00
Thyroid disease	1039 (1.6)	139 (2.0)	0.02
Chronic viral hepatitis and HIV	197 (0.3)	54 (0.8)	0.06
Rheumatic disease	235 (0.4)	55 (0.8)	0.05
Surgery^e^	≤ 5	≤ 5	≤ 0.02
Musculoskeletal pain^e^	20 (0.0%)	12 (0.2)	0.04
*Pre‐pregnancy maternal medicine use (year prior to LMP)*			
Metformin	742 (1.2)	148 (2.1)	0.07
Non‐opioid analgesics	8944 (14.0)	2179 (30.6)	0.41
Gabapentinoids	15 (0.0)	11 (0.2)	0.04
Opioids^f^	6445 (10.1)	2451 (34.4)	0.61
Psychotropics	1978 (3.1)	756 (10.6)	0.30
Pregnancy category D/X medicines^g^	2110 (3.3)	455 (6.4)	0.14
Systemic corticosteroids	2653 (4.2)	667 (9.4)	0.21
*Pre‐pregnancy maternal healthcare utilisation (year prior to LMP)*			
Number of hospital admissions			
0	47 877 (70.6)	4816 (62.8)	0.08
1	14 600 (21.5)	1820 (23.7)	0.02
≥ 2	5365 (7.9)	1028 (13.4)	0.06
Number of ED presentations			
0	54 808 (80.8)	5414 (70.6)	0.10
1	8039 (11.8)	1159 (15.1)	0.03
≥ 2	4995 (7.4)	1091 (14.2)	0.07
Number of GP visits			
0	32 917 (51.6)	2778 (39.0)	0.26
1	15 988 (25.1)	1762 (24.8)	0.01
≥ 2	14 861 (23.3)	2576 (36.2)	0.29

Abbreviations: ED, emergency department; GP, general practitioners; HIV, human immunodeficiency virus; SD, standard deviation.

^a^ Suppressed to prevent calculations of cells with small count.

^b^ Social security benefits refer to government‐administered financial assistance provided to eligible individuals.

^c^ Parental characteristics, collected at initial NSW government school enrolment (typically before third grade), included education level and occupation of mother and second parent (if available).

^d^ Occupation group is completed by parents of children applying to enrol in a NSW government school for the first time. Examples of professionals listed in each group include: Group 1: elected officials and senior managers. Group 2: business managers and associate professors. Group 3: tradespeople and advanced/intermediate clerical staff. Group 4: machine operators, assistances, sales and service staff.

^e^ Ascertained in the year prior to LMP until LMP.

^f^ Ascertained in the year prior to LMP until 90 days before LMP.

^g^ Ascertained during pregnancy.

### 3.1 Differences in Academic Performance

**FIGURE 2:**
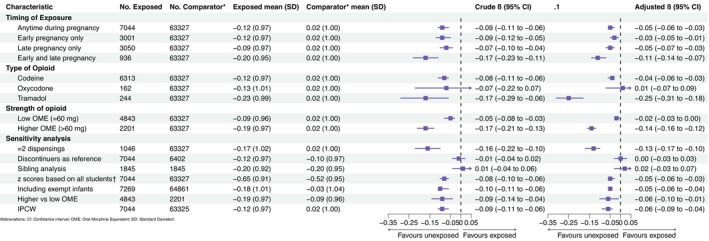
Differences in mean z scores in reading between third‐grade children with and without prenatal opioid analgesic exposure: Crude and adjusted beta coefficients (β) with 95% confidence intervals (CI). Covariates included in the adjusted analyses included child's sex, child's year of birth, test year, language spoken at home, maternal age, born in western country, does not have a partner, social security benefits received, previous caesarean delivery, smoking during pregnancy, area‐based socioeconomic disadvantage, area‐based remoteness, multifetal gestation, parental highest educational attainment, parent occupation, maternal conditions, pre‐pregnancy maternal medicine use, and pre‐pregnancy healthcare utilisation. *Comparator reference groups consisted of children that were not prenatally exposed to opioid analgesics, with three exceptions: (1) For discontinuers, the reference group included children prenatally exposed in the 90 days before the last menstrual period (LMP) but not during pregnancy; (2) for the sibling analysis, the reference group comprised of siblings with no prenatal opioid exposure; and (3) for the OME comparison, low OME exposure was used as the reference group. ^†^z scores based on both concessional and general beneficiaries' test scores.

**FIGURE 3:**
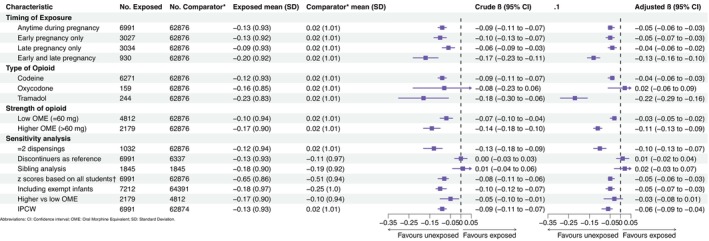
Differences in mean z scores in numeracy between third‐grade children with and without prenatal opioid analgesic exposure: Crude and adjusted beta coefficients (β) with 95% confidence intervals (CI). Covariates included in the adjusted analyses included child's sex, child's year of birth, test year, language spoken at home, maternal age, born in western country, does not have a partner, social security benefits received, previous caesarean delivery, smoking during pregnancy, area‐based socioeconomic disadvantage, area‐based remoteness, multifetal gestation, parental highest educational attainment, parent occupation, maternal conditions, pre‐pregnancy maternal medicine use, and pre‐pregnancy healthcare utilisation. *Comparator reference groups consisted of children that were not prenatally exposed to opioid analgesics, with three exceptions: (1) For discontinuers, the reference group included children prenatally exposed in the 90 days before the last menstrual period (LMP) but not during pregnancy; (2) for the sibling analysis, the reference group comprised of siblings with no prenatal opioid exposure; and (3) for the OME comparison, low OME exposure was used as the reference group. ^†^z scores based on both concessional and general beneficiaries' test scores.

Compared with unexposed children, prenatal exposure to any opioid analgesic was associated with a slight decrease in standardised reading and numeracy scores adjusted mean difference β [aβ] −0.05 (95% CI −0.06 to −0.03 for both), below the conventional threshold for a small effect (Cohen's *d* = 0.2) [31]. Similar differences were observed among children exposed to codeine (aβ −0.04, 95% CI −0.06 to −0.03 for both reading and numeracy), and among those exposed to oxycodone (aβ −0.01, 95% CI −0.07 to 0.09 for reading; aβ −0.02, 95% CI −0.06 to 0.09 for numeracy) (Figures 2 and 3).

Larger magnitude differences were observed among children exposed to tramadol, with reading aβ −0.25 (95% CI: −0.31 to −0.18) and numeracy aβ −0.22 (95% CI: −0.29 to −0.16), exceeding the conventional threshold for a small effect. Differences of intermediate magnitude were observed for exposure during both early and late (reading aβ −0.11, 95% CI: −0.14 to −0.07; numeracy aβ −0.13, 95% CI: −0.16 to −0.10) and for higher OME doses (reading aβ −0.14, 95% CI: −0.16 to −0.12; numeracy aβ −0.11, 95% CI: −0.13 to −0.09).

### 3.2 Secondary Outcome

Similar patterns to the primary outcome were observed for secondary outcomes based on scores below the NMS threshold, with the highest relative risks (RR) observed among children exposed in both early and late pregnancy (reading 1.20, 95% CI 0.98–1.46; numeracy 1.16, 95% CI 0.92–1.44), and to tramadol (reading 1.20, 95% CI 0.85–1.70; numeracy 1.08, 95% CI 0.74–1.58) (Figures S6 and S7). However, the confidence intervals for these estimates are wide and compatible with both modest increases and decreases in risk.

### 3.3 Sensitivity Analysis

Compared to the primary analysis, there was a slightly larger difference between mean z‐scores when prenatal exposure was redefined as at least two dispensings (reading aβ −0.13, 95% −0.17 to −0.10, numeracy aβ −0.10, 95% −0.13 to −0.07). For discontinuer and sibling analyses, results were close to the null for any opioid, codeine, or oxycodone exposure, though confidence intervals were wider for individual opioids. For tramadol, estimates were only slightly attenuated, although confidence intervals were wide (reading: aβ −0.17, 95% CI −0.26 to −0.08; numeracy: aβ −0.18, 95% CI −0.27 to −0.09) (Figure S8). Results were consistent in the analysis based on z‐scores from test results of all students (general and concessional), as well as in our analyses accounting for potential selection bias (Figures 2 and 3; Figures S6 and S7).

### 3.4 Post Hoc Analyses

The association between higher OME exposure and poor academic performance was attenuated when compared to low OME exposure (Figures 2 and 3; Figures S7 and S8). The *E*‐value indicates an unmeasured confounder would need a risk ratio of 1.82 (95% CI: 1.36–2.58) with both tramadol exposure and academic performance to fully explain away the observed association. We found similar standardised proportions of adverse birth outcomes between the exposed and unexposed groups, suggesting these outcomes likely play a minimal role in the relationship between opioid exposure and academic performance (Table S7).

## 4 Discussion

### 4.1 Main Findings

In this population‐based cohort study of children born to concessional beneficiaries in NSW, Australia, prenatal exposure to opioid analgesics across various pregnancy periods, doses, and monotherapies, with the potential exception of tramadol, was associated with small differences in third grade reading and numeracy skills. The consistency of the small effect sizes across primary and secondary outcomes, especially when considered relative to the plausible magnitude of residual bias, together with the near‐null values in sensitivity analyses with improved confounding control, suggests that prenatal opioid exposure is unlikely to have clinically meaningful impacts on academic performance. Compared with other prenatal exposures, the effects we observed for most opioid exposures (*β* ~0.05) were small. By comparison, prenatal exposure to established teratogens such as valproate has been linked to markedly lower standardized language and mathematics scores in adolescence (*β* −0.27 to −0.33), whereas safer alternatives like lamotrigine showed effects similar to those we observed [32]. Collectively, these findings indicate that, aside from uncertainty around tramadol, prenatal opioid exposure is unlikely to meaningfully impact academic outcomes.

### 4.2 Interpretation

While tramadol was the only exposure to exceed our predefined threshold for a meaningful academic difference, this finding should be interpreted in the context of additional evidence. First, our discontinuer and sibling analyses suggest that unmeasured confounding factors may partially account for the association with academic performance. Second, post hoc sensitivity analyses indicate that even a modest confounder could account for the observed effect. Third, the higher median tramadol dose compared to other opioids raises the possibility of a dose–response relationship or confounding by indication. Fourth, the effect sizes for the secondary outcome were small and inconsistent, although confidence intervals were wider due to smaller group sizes. Fifth, although tramadol is unique in acting as both a μ‐opioid receptor agonist and a serotonin/norepinephrine reuptake inhibitor, previous research has not demonstrated any association between academic performance and prenatal exposure to antidepressants with similar reuptake inhibition properties [33, 34]. Taken together, these results raise the possibility that unmeasured confounding factors specifically associated with prenatal tramadol exposure may have influenced our findings. In our data, women prescribed tramadol exhibited higher rates of mental health conditions, analgesic use, and exposure to teratogenic medicines compared to unexposed women and codeine users, suggesting greater pain burden and more complex health profiles. However, similar patterns were observed among those who used oxycodone, meaning women that used tramadol would need to differ in some unmeasured way to explain the tramadol‐specific effects. Although we adjusted for a broad range of measured covariates, we were unable to account for factors such as indication for treatment, or residual confounding from under‐ascertained maternal conditions, which may vary across opioid types. Future studies incorporating more detailed clinical information may help to disentangle these relationships. As unmeasured confounding cannot be definitively excluded, these findings should be interpreted as a potential signal warranting further investigation rather than as evidence of a causal association.

Our findings regarding prenatal exposure to any opioid analgesic are consistent with those of a previous Norwegian cohort study of fifth‐grade children, although it should be noted that both sets of results likely reflect the effect of codeine exposure given its predominance in both study populations [8]. Our research expands the evidence on long‐term safety of analgesic opioids used during pregnancy by evaluating academic performance related to commonly used opioids worldwide, including tramadol [4]. Our findings primarily provide evidence of safety for acute and low‐dose opioid exposure during pregnancy, and they may not be generalizable to contexts involving higher dosages or repeated opioid dispensings, particularly given some evidence of potential dose–response and duration effects.

### 4.3 Strengths and Limitations

The primary strength of this study is the use of real‐world, population‐level data to understand the impact of prenatal opioid exposure on academic performance among children. Our study has several limitations. First, dispensing records may not accurately reflect actual use. However, our sensitivity analysis restricting to ≥ 2 dispensings yielded similar results, increasing our confidence that our exposure measure reasonably approximates actual use. Second, limited exposure data prevented sensitivity analyses requiring ≥ 2 dispensings. This constraint may have contributed to some of the observed null associations through exposure misclassification. Third, our data do not capture medicines supplied in public hospitals, privately funded prescriptions, or over the counter (OTC) low‐dose codeine (≤ 15 mg), which was available until February 2018 [35]. Privately dispensed opioids likely had minimal impact on our findings given that our concessional beneficiary population would have preferentially accessed lower‐cost, subsidised medicines. However, incomplete capture of codeine use may have resulted in exposure misclassification, potentially biasing estimates toward the null. In 2014, privately funded and OTC codeine comprised approximately one‐fifth of total opioid utilisation in Australia [36]. Fourth, we restricted our study population to children of concessional beneficiaries to ensure complete capture of medicine dispensings. Consequently, compared to the general population, mothers in our sample were disproportionally younger, had more co‐morbidities and experienced greater socio‐economic disadvantage (Table S8). These differences mean that the observed associations are most directly applicable to women with similar clinical and socioeconomic characteristics. In populations with lower comorbidity burden or higher socioeconomic status, the magnitude of the associations could differ due to differences in baseline risk factors, and this should be considered when interpreting or generalising the findings. Fifth, selection bias may have influenced our findings, as approximately 17% of eligible children lacked test scores due to non‐participation in tests or loss to follow‐up. However, we believe our findings are robust because: (1) proportions of exposed and unexposed children were similar across missing data categories, (2) children lost to follow‐up were largely comparable to those with test scores, and (3) sensitivity analyses incorporating exempt children and applying inverse probability of censoring weights yielded results consistent with our primary analysis. Finally, we lacked information on clinical indications for opioid use, potentially introducing confounding by indication which could bias results away from the null if the underlying conditions are independently associated with poorer academic outcomes.

## 5 Conclusions

Overall, our findings suggest that prenatal exposure to opioid analgesics, with the possible exception of tramadol, does not have an academically meaningful impact on third‐grade academic performance. However, whether the observed tramadol results indicate a causal relationship or reflect unmeasured confounding remains unclear, warranting further investigation. These long‐term safety findings should be considered alongside evidence of short‐term risks when assessing the benefits and risks of prescribing opioids during pregnancy.

## Author Contributions

Dr. Bianca Varney designed the study, planned and executed analysis, drafted the initial manuscript, and revised the manuscript. A/Prof Jonathan Brett conceptualised the study, contributed to the design and interpretation of the results, and critically reviewed and revised the manuscript for important intellectual content. Prof Helga Zoega conceptualised the study, contributed to the design and interpretation of the results, and critically reviewed and revised the manuscript for important intellectual content. Dr. Malcolm B. Gillies contributed to the design, analysis, and interpretation of results, and had input in the initial manuscript and critically reviewed and revised the manuscript for important intellectual content. Dr. Claudia Bruno and Prof Natasha Nassar contributed to the design and interpretation of the results, critically reviewed and revised the manuscript for important intellectual content. Dr. Antonia Shand contributed to the interpretation of the results, critically reviewed and revised the manuscript for important intellectual content. A/Prof Alys Havard critically reviewed and revised the manuscript for important intellectual content. All authors approved the final manuscript as submitted and agree to be accountable for all aspects of the work.

## Funding

This research was supported by the National Health and Medical Research Council (NHMRC) Centre of Research Excellence in Medications Intelligence (ID: 1196900) and an NHMRC Investigator Grant (ID: 1196560). Prof Helga Zoega was supported by a UNSW Scientia Program Award during the conduct of the study. Prof Natasha Nassar is supported by NHMRC Investigator Grant (APP1197940) and the Financial Markets Foundation for Children.

## Conflicts of Interest

The Medicines Intelligence Research Program, School of Population Health, UNSW has received research funding from IQVIA Australia, unrelated to this project. The remaining authors declare no conflicts of interest.

## Supporting information


**Figure S1:** Graphical depiction of the study design.
**Figure S2:** Directed Acyclic Graph of the total effect of prenatal opioid exposure on academic performance. The diagram displays measured and adjusted factors (white) with closed (adjusted) confounding pathways (black lines), unmeasured and not adjusted for intermediate variables (blue). Causal pathways are denoted by green lines.
**Figure S3:** Frequency and proportion of prenatal opioid analgesic dispensings among exposed children, stratified by opioid type and categorised by the number of dispensings (1, 2, or ≥ 3).
**Figure S4:** Boxplot showing total OME among children prenatally exposed to opioid analgesics, stratified by opioid type.
**Figure S5:** Absolute standardised mean differences in baseline characteristics between children prenatally exposed to opioids or unexposed, before and after applying propensity score weights. Values closer to zero indicate better covariate balance. The dotted line represents the conventional thresholds of ±0.1 for acceptable balance. Absolute standardised mean differences for respiratory disease, chronic liver disease, cancer diagnosis or treatment, and surgery were omitted in accordance with data suppression for small cell sizes.
**Figure S6:** Relative risk (95% confidence interval) of scoring below national minimum standards for reading after prenatal opioid analgesic exposure: Risk estimates by timing, monotherapy type, opioid dose, and sensitivity analyses. Covariates included in the adjusted analyses included child's sex, child's year of birth, test year, language spoken at home, maternal age, born in western country, does not have a partner, social security benefits received, previous caesarean delivery, smoking during pregnancy, area‐based socioeconomic disadvantage, area‐based remoteness, multifetal gestation, parental highest educational attainment, parent occupation, maternal conditions, pre‐pregnancy maternal medicine use, and pre‐pregnancy healthcare utilisation.
**Figure S7:** Relative risk (95% confidence interval) of scoring below national minimum standards for numeracy after prenatal opioid analgesic exposure. Risk estimates by timing, monotherapy type, opioid dose, and sensitivity analyses. Covariates included in the adjusted analyses included child's sex, child's year of birth, test year, language spoken at home, maternal age, born in western country, does not have a partner, social security benefits received, previous caesarean delivery, smoking during pregnancy, area‐based socioeconomic disadvantage, area‐based remoteness, multifetal gestation, parental highest educational attainment, parent occupation, maternal conditions, pre‐pregnancy maternal medicine use, and pre‐pregnancy healthcare utilisation.
**Figure S8:** Differences in mean z‐scores in reading and numeracy among third‐grade children among children of discontinuers or unexposed siblings and among children prenatally exposed to opioid monotherapies. Crude and adjusted beta coefficients (*β*) with 95% confidence intervals (CI). Covariates included in the adjusted analyses included child's sex, child's year of birth, test year, language spoken at home, maternal age, born in western country, does not have a partner, social security benefits received, previous caesarean delivery, smoking during pregnancy, area‐based socioeconomic disadvantage, area‐based remoteness, multifetal gestation, parental highest educational attainment, parent occupation, maternal conditions, pre‐pregnancy maternal medicine use, and pre‐pregnancy healthcare utilisation. *Comparator reference groups consisted of children that were not prenatally exposed to opioid analgesics, with three exceptions: (1) For discontinuers, the reference group included children prenatally exposed in the 90 days before the last menstrual period (LMP) but not during pregnancy; (2) for the sibling analysis, the reference group comprised of siblings with no prenatal opioid exposure; and (3) for the OME comparison, low OME exposure was used as the reference group.
**Table S1:** Exclusion criteria
**Table S2:** Prescription opioid analgesics included in the study and their corresponding Anatomical Therapeutic Chemical (ATC) Classification codes.
**Table S3:** List of covariates, relevant databases, ascertainment window and codes used for their identification.
**Table S4:** List of birth outcomes, data sources and codes used for their identification
**Table S5:** Comparison of children characteristics of those with test scores and those classified as lost to follow‐up.
**Table S6:** Maternal and child characteristics by exposure to specific opioid analgesics during pregnancy, for children of concessional beneficiaries between January 01, 2003, to March 26, 2011.
**Table S7:** Crude and standardised proportions of birth outcomes per 100 infants with 95% confidence intervals among those with prenatal opioid exposure and those unexposed.
**Table S8:** Comparison of characteristics of children of eligible concessional beneficiaries (study population) and those born to women that did not meet the continous concessional beneficiary status.
**Table S9:** STROBE Statement—Checklist of items that should be included in reports of cohort studies.

## Data Availability

The data sets were constructed with the permission of each of the source data custodians and with specific ethical approvals. The authors do not have permission to share patient‐level data because of the highly confidential nature of the data. Permission to access to the data is restricted to researchers named and approved by relevant Human Research Ethics Committees.
