# Supplementary material for: Prenatal Prescription Opioid Analgesic Exposure and Academic Performance in Third Grade Children: A Population‐Based Cohort Study
Source: BJOG. 2026 Mar 15;133(8):1658–70. doi: 10.1111/1471-0528.70221 (PMC13254002; doi:10.1111/1471-0528.70221)
Supplement: Supplementary file 1 — Figure S1: Graphical depiction of the study design. Figure S2: Directed Acyclic Graph of the total effect of prenatal opioid exposure on academic performance. The diagram displays measured and adjusted factors (white) with closed (adjusted) confounding pathways (black lines), unmeasured and not adjusted for intermediate variables (blue). Causal pathways are denoted by green lines. Figure S3: Frequency and proportion of prenatal opioid analgesic dispensings among exposed children, stratified by opioid type and categorised by the number of dispensings (1, 2, or ≥ 3). Figure S4: Boxplot showing total OME among children prenatally exposed to opioid analgesics, stratified by opioid type. Figure S5: Absolute standardised mean differences in baseline characteristics between children prenatally exposed to opioids or unexposed, before and after applying propensity score weights. Values closer to zero indicate better covariate balance. The dotted line represents the conventional thresholds of ±0.1 for acceptable balance. Absolute standardised mean differences for respiratory disease, chronic liver disease, cancer diagnosis or treatment, and surgery were omitted in accordance with data suppression for small cell sizes. Figure S6: Relative risk (95% confidence interval) of scoring below national minimum standards for reading after prenatal opioid analgesic exposure: Risk estimates by timing, monotherapy type, opioid dose, and sensitivity analyses. Covariates included in the adjusted analyses included child's sex, child's year of birth, test year, language spoken at home, maternal age, born in western country, does not have a partner, social security benefits received, previous caesarean delivery, smoking during pregnancy, area‐based socioeconomic disadvantage, area‐based remoteness, multifetal gestation, parental highest educational attainment, parent occupation, maternal conditions, pre‐pregnancy maternal medicine use, and pre‐pregnancy healthcare utilisation. Figure S7: Relat [file BJO-133-1658-s001.docx]

**Supplementary material:**

***Prenatal prescription opioid analgesic exposure and academic performance in third grade children***

**Appendix:**

[Appendix 1: Detailed description of data sources used in this study: 2](#_Toc200392826)

[Appendix 2: Detailed description of methods for the IPCW 3](#_Toc200392827)

**Figures:**

[Figure S1: Graphical depiction of the study design 4](#_Toc200392845)

[Figure S2: Directed Acyclic Graph of possible causal pathways relevant to prenatal opioid analgesic exposure academic performance. 5](#_Toc200392846)

[Figure S3: Frequency and proportion of prenatal opioid analgesic dispensings among exposed children, stratified by opioid type and categorised by the number of dispensings (1, 2, or ≥3). 6](#_Toc200392847)

[Figure S4: Boxplot showing total OME among children prenatally exposed to opioid analgesics, stratified by opioid type. 7](#_Toc200392848)

[Figure S5: Absolute standardised mean differences in baseline characteristics between children prenatally exposed to opioids or unexposed, before and after applying propensity score weights 8](#_Toc200392849)

[Figure S6: Relative risk (95% confidence interval) of scoring below national minimum standards for reading after prenatal opioid analgesic exposure: Risk estimates by timing, monotherapy type, opioid dose, and sensitivity analyses. 9](#_Toc200392850)

[Figure S7: Relative risk (95% confidence interval) of scoring below national minimum standards for numeracy after prenatal opioid analgesic exposure. Risk estimates by timing, monotherapy type, opioid dose, and sensitivity analyses.. 10](#_Toc200392851)

[Figure S8: Differences in mean z-scores in reading and numeracy among third-grade children among children of discontinuers or unexposed siblings and among children prenatally exposed to opioid monotherapies. Crude and adjusted beta coefficients (β) with 95% confidence intervals (CI). 11](#_Toc200392852)

**Tables:**

[Table S1: Exclusion criteria 12](#_Toc200392939)

[Table S2: Prescription opioid analgesics included in the study and their corresponding Anatomical Therapeutic Chemical (ATC) Classification codes. 13](#_Toc200392940)

[Table S3: List of covariates, relevant databases, ascertainment window and codes used for their identification. 14](#_Toc200392941)

[Table S4: List of birth outcomes, data sources and codes used for their identification 22](#_Toc200392942)

[Table S5: Comparison of children characteristics of those with test scores and those classified as lost to follow-up. 26](#_Toc200392943)

[Table S6: Maternal and child characteristics by exposure to specific opioid analgesics during pregnancy, for children of concessional beneficiaries between January 01, 2003, to March 26, 2011. 28](#_Toc200392944)

[Table S7: Crude and standardised proportions of birth outcomes per 100 infants with 95% confidence intervals among those with prenatal opioid exposure and those unexposed. 32](#_Toc200392945)

[Table S8: Comparison of characteristics of children of eligible concessional beneficiaries (study population) and those born to women that did not meet the continous concessional beneficiary status. 33](#_Toc200392946)

[Table S9: STROBE Statement—Checklist of items that should be included in reports of *cohort studies* 35](#_Toc200392947)

Appendix 1: Detailed description of data sources used in this study:

**Perinatal Data Collection (PDC):** the PDC is a mandatory reporting system that captures all births in NSW, including both live births and stillbirths, where the pregnancy reached at least 20 weeks gestation, or the baby weighed at least 400g. It contains detailed information about maternal characteristics, pregnancy complications, and birth outcomes, collected by healthcare providers at the time of birth.

**Pharmaceutical Benefits Scheme (PBS):** the PBS contains records for all PBS-listed medicines for which the Commonwealth pays a subsidy. Australia has a universal healthcare system providing subsidised health services to Australian citizens, permanent residents, and residents from countries with reciprocal healthcare agreements, including access to subsidised prescription medicines through the PBS. The Australian Federal government subsidises PBS-listed medicines when their prices exceed co-payment thresholds. Concessional beneficiaries – including social security recipients, low-income earners, age pensioners, individuals with disabilities, and caregivers – pay lower co-payments ($7.70 in 2024) than general beneficiaries ($31.60), with approximately 25% of individuals accessing the PBS holding concessional status (1, 2). The prices of many common medicines are below the general co‐payment threshold, but all are above the concessional co‐payment threshold. Prior to July 2012, only medicines that cost above the co-payment threshold were recorded, and ascertained of PBS medicines was complete only for concessional beneficiaries. Since July 2012, all PBS-listed medicines have been captured in PBS dataset, regardless of cost. The PBS does not include private prescriptions, over-the-counter medicines, or medicines prescribed during public hospital stays.

**The National Assessment Program – Literary and Numeracy (NAPLAN):** the NAPLAN data consists of standardised tests administered to all students in NSW in Years 3, 5, 7, and 9. These tests assess literacy, writing, language conventions, and numeracy skills.

**Social security data – Data Over Multiple Individual Occurrences (DOMINO):** the DOMINO data provides records of individuals' interactions with Department of Social Services (DSS) welfare programs over time, providing information on welfare recipients, including their demographics, benefit history, concessions, education (where available), and housing circumstances.

**NSW Admitted Patient Data Collection (NSW APDC):** the APDC provides records of all inpatient separations (discharges, transfers and deaths) in NSW hospitals, including public, private, psychiatric and repatriation hospitals in NSW, as well as public multi-purpose services, private day procedure centres and public nursing homes. It captures detailed information about diagnoses, procedures, and length of stay for all hospital admissions.

**NSW Emergency Department Data Collection (NSW EDDC):** the EDDC captures patient presentations to emergency departments (EDs) in NSW public hospitals and participating private hospitals. Each record represents an emergency department presentation for assessment or treatment, including patients transferred from other hospital units or facilities.

**NSW Controlled Drugs Data Collection (CoDDaC):** CoDDaC data contains information on authorities to prescribe Schedule 8 controlled drugs in NSW, including opioids used in opioid agonist therapy (OAT). The data contains detailed information on prescribers, recipients, and medicine information.

**Mortality data – Registry of Births, Deaths and Marriages (RBDM):** RBDM data captures all deaths registered in NSW.

Appendix 2: Detailed description of methods for the IPCW

We applied inverse-probability of censoring weights (IPCW) alongside the propensity score weights in each model to adjust for potential attrition bias arising from children with missing test scores. This adjustment excluded children missing due to death or administrative censoring. We predicted the probability of missing test results using maternal sociodemographic characteristics, child's sex, year of birth, and additional covariates including whether the child had medical services subsidised by the government [Medicare Benefits Schedule (MBS)] or medicine dispensings outside of NSW, had a diagnosis of attention-deficit/hyperactivity disorder (ADHD) (defined as a dispensings of an ADHD medicine, Anatomical Therapeutic Chemical code N06BA04, N06BA04, N06BA02, N06BA12, N06BA09, C02AC02, C02AC01), received any psychotropic medicine prior to the age of 9 years (see medicine list in Table S3) or child morbidities identified using the Paediatric Comorbidity Index (3) with adaptations for the Australian context as detailed in eBox 1. These weights were stabilized and trimmed by removing observations with extreme weights (i.e., percentile < 1% and > 99%).

Box 1: Modifications to the original paediatric morbidity score for the Australia setting

| **Condition** | **ICD-10-AM Code Modifications** |
| --- | --- |
| **Alcohol Abuse** | Added: Z71.4, Z72.1 |
| **Cancer** | Added: Z51.1 |
| **Asthma** | Added: J46, U83.3 |
| **Cardiovascular** | Added: U82.1, U82.2 |
| **Depression** | Added: F06.233, U79.3 Removed: F06.32, F06.31, F34.9 |
| **Developmental Disorder** | Removed: H93.25 |
| **Diabetes** | Removed: E08–E09 Added: E14 |
| **Drug Abuse** | Added: Z50.2, Z50.3, Z72.2 |
| **Epilepsy** | Added: G41, F80.3 |
| **Psychotic Disorders** | Added: F06.31, F06.32, U79.2 Removed: F06.33 |
| **Smoking** | Added: Z72.0, Z86.43 Removed: Z87.891 |

Abbreviations: ICD-10-AM – International Classification of Diseases, 10th Revision, Australian Modification.

,

**
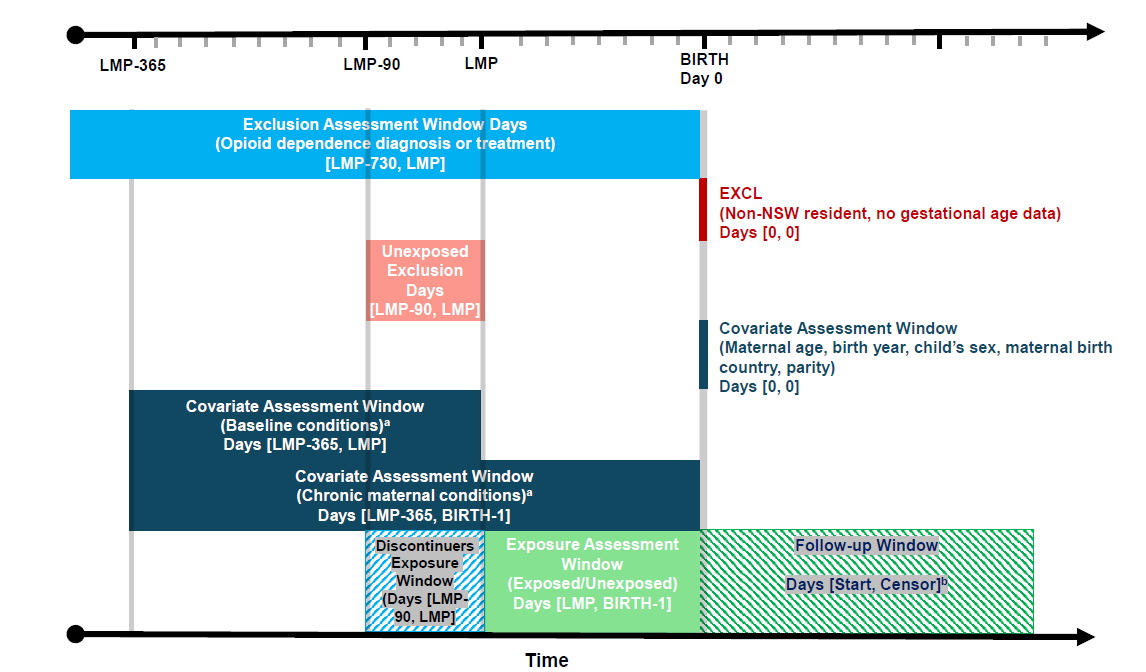
**

Figure S1: Graphical depiction of the study design
a. Full list and code algorithms provided in Table S3;
b. Follow up time started on the date of childbirth and ended at the earliest of an outcome, death, or end of the study period.


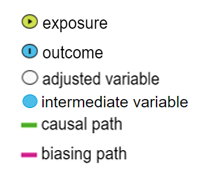

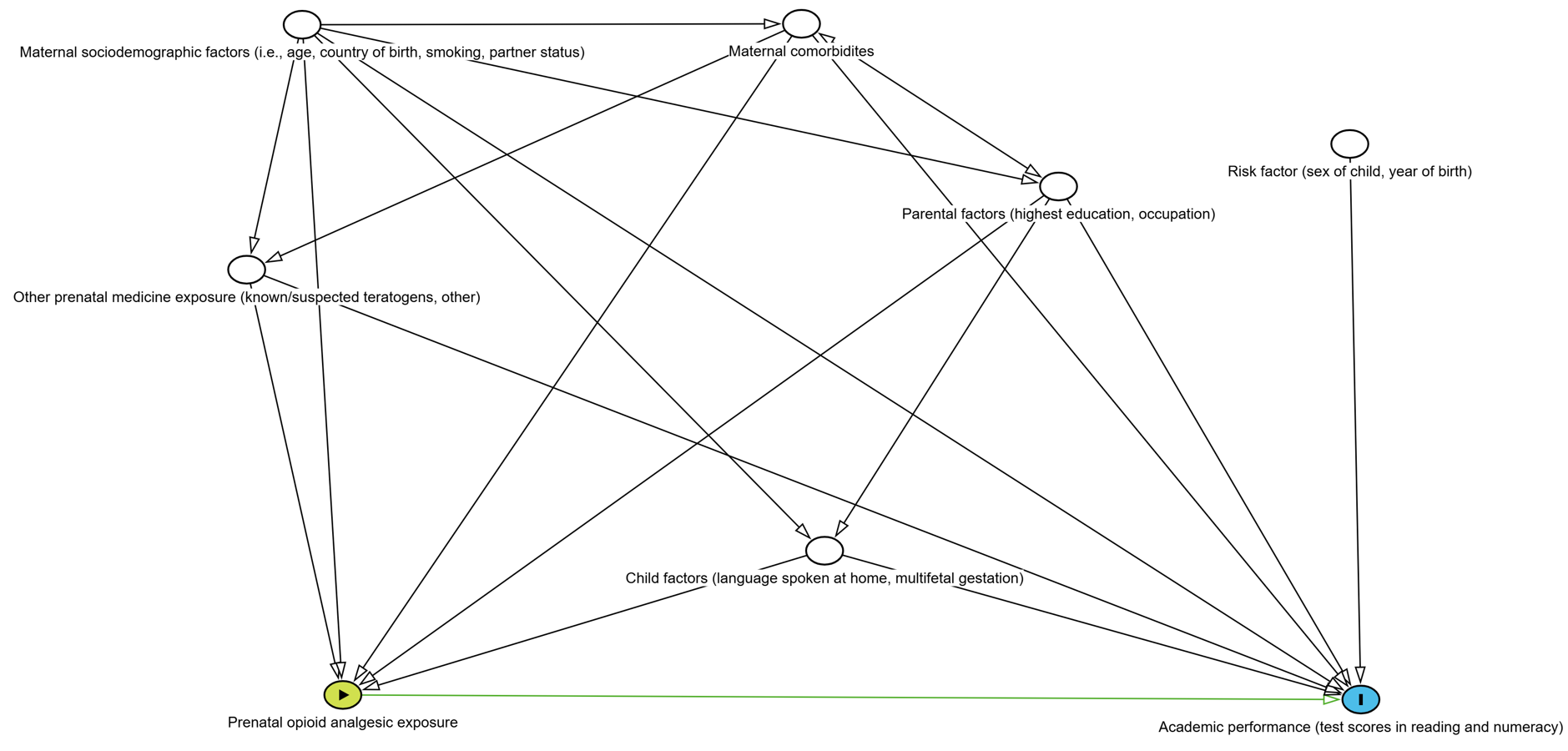


Figure S2: Directed Acyclic Graph of the total effect of prenatal opioid exposure on academic performance. The diagram displays measured and adjusted factors (white) with closed (adjusted) confounding pathways (black lines), unmeasured and not adjusted for intermediate variables (blue). Causal pathways are denoted by green lines.


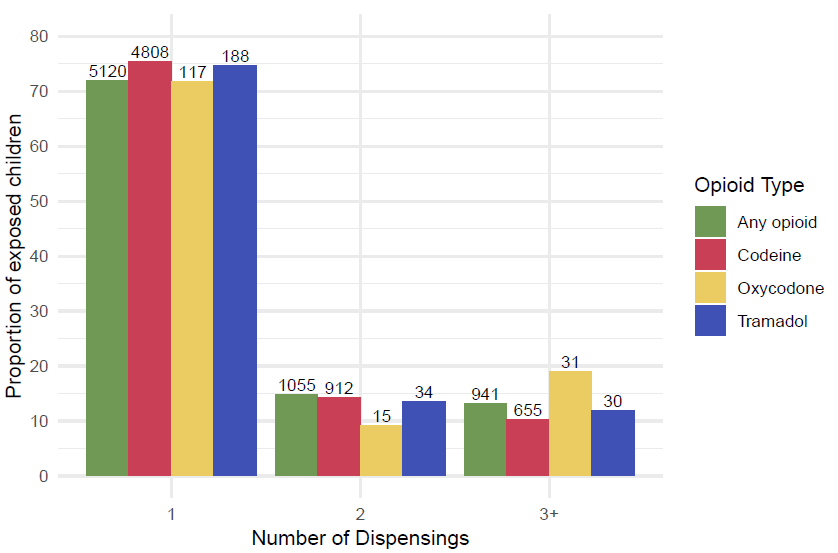


Figure S3: Frequency and proportion of prenatal opioid analgesic dispensings among exposed children, stratified by opioid type and categorised by the number of dispensings (1, 2, or ≥3).

**
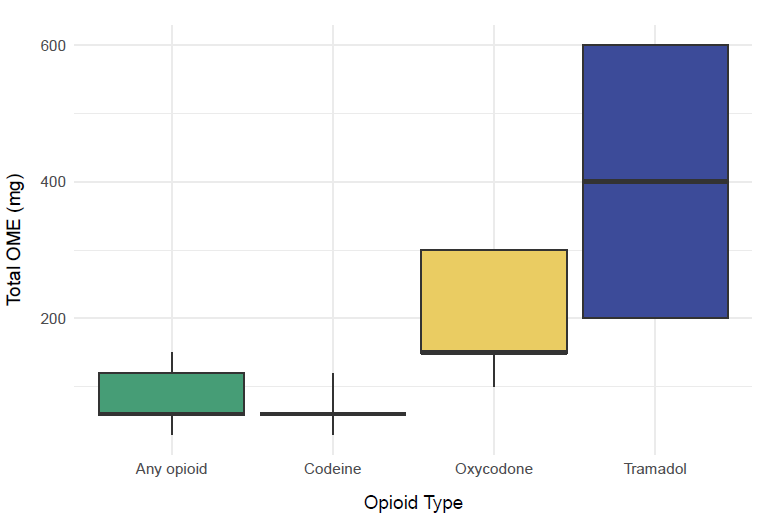
**

Figure S4: Boxplot showing total OME among children prenatally exposed to opioid analgesics, stratified by opioid type.


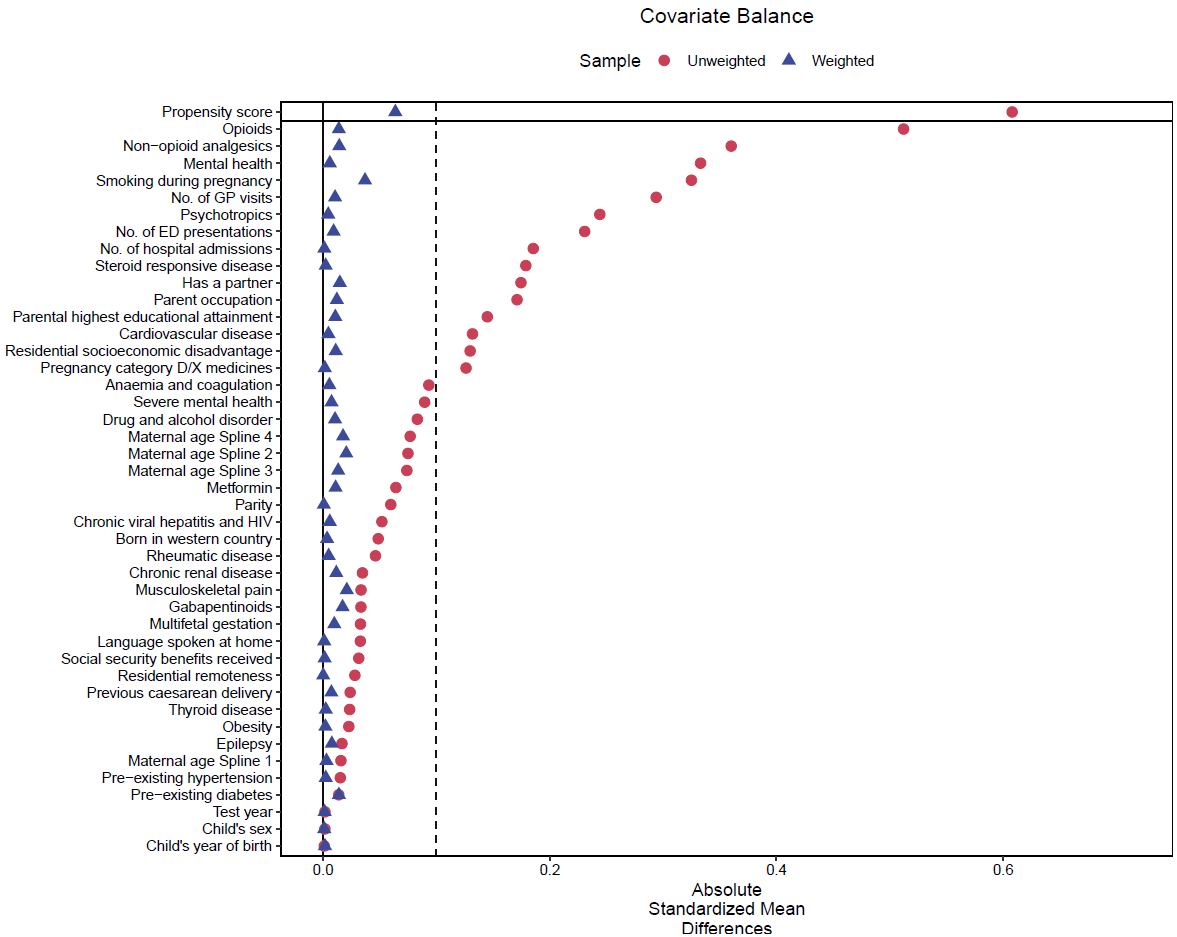


Figure S5: Absolute standardised mean differences in baseline characteristics between children prenatally exposed to opioids or unexposed, before and after applying propensity score weights. Values closer to zero indicate better covariate balance. The dotted line represents the conventional thresholds of ±0.1 for acceptable balance. Absolute standardized mean differences for respiratory disease, chronic liver disease, cancer diagnosis or treatment, and surgery were omitted in accordance with data suppression for small cell sizes.


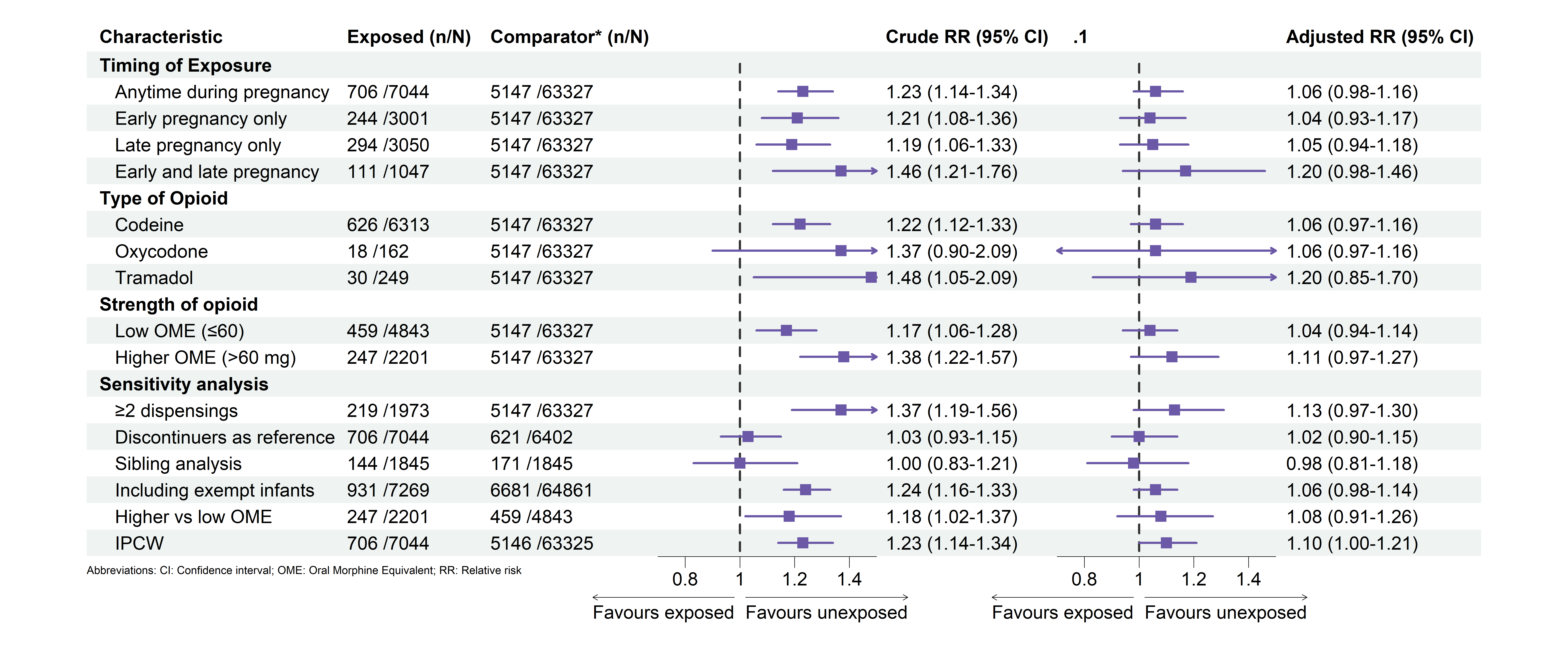


Figure S6: Relative risk (95% confidence interval) of scoring below national minimum standards for reading after prenatal opioid analgesic exposure: Risk estimates by timing, monotherapy type, opioid dose, and sensitivity analyses. Covariates included in the adjusted analyses included child’s sex, child’s year of birth, test year, language spoken at home, maternal age, born in western country, does not have a partner, social security benefits received, previous caesarean delivery, smoking during pregnancy, area-based socioeconomic disadvantage, area-based remoteness, multifetal gestation, parental highest educational attainment, parent occupation, maternal conditions, pre-pregnancy maternal medicine use, and pre-pregnancy healthcare utilisation.

*Comparator reference groups consisted of children that were not prenatally exposed to opioid analgesics, with three exceptions: (1) For discontinuers, the reference group included children prenatally exposed in the 90 days before the last menstrual period (LMP) but not during pregnancy; (2) for the sibling analysis, the reference group comprised siblings with no prenatal opioid exposure; and (3) for the OME comparison, low OME exposure was used as the reference group.


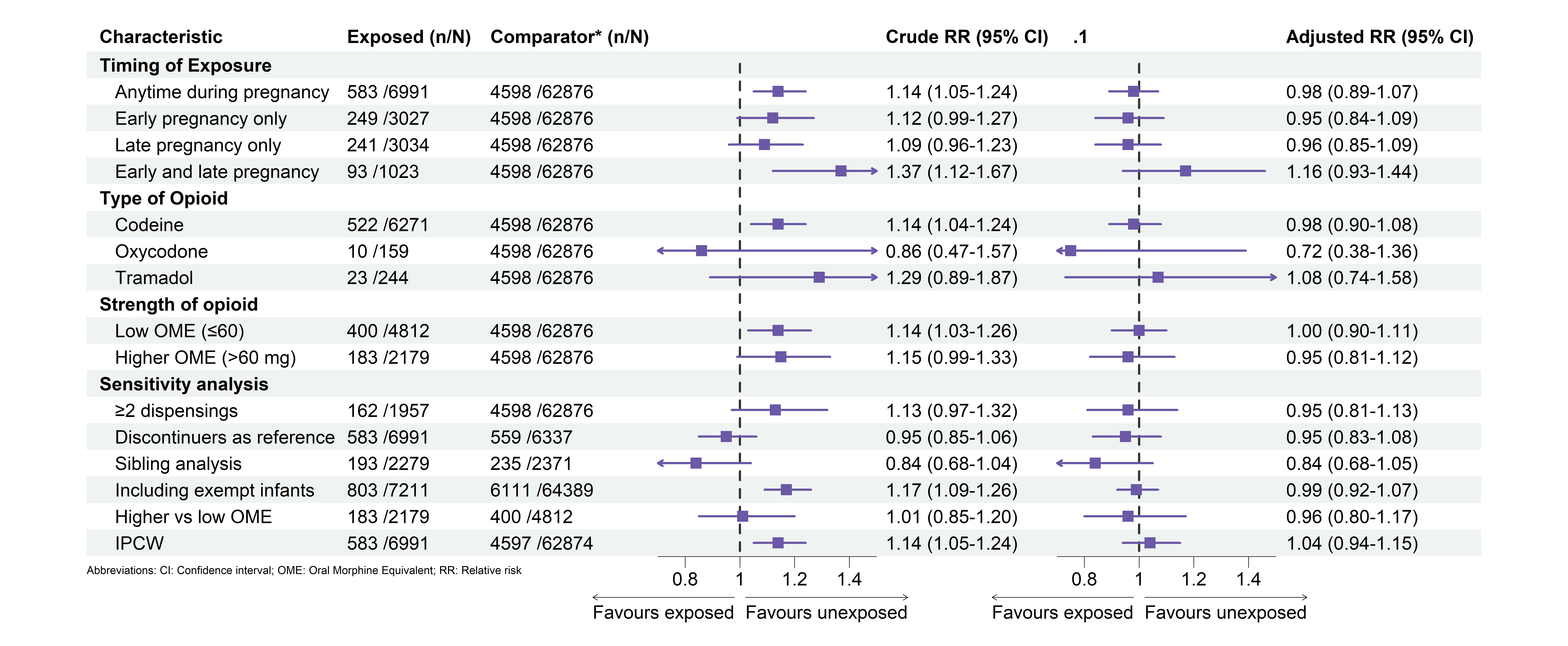


Figure S7: Relative risk (95% confidence interval) of scoring below national minimum standards for numeracy after prenatal opioid analgesic exposure. Risk estimates by timing, monotherapy type, opioid dose, and sensitivity analyses. Covariates included in the adjusted analyses included child’s sex, child’s year of birth, test year, language spoken at home, maternal age, born in western country, does not have a partner, social security benefits received, previous caesarean delivery, smoking during pregnancy, area-based socioeconomic disadvantage, area-based remoteness, multifetal gestation, parental highest educational attainment, parent occupation, maternal conditions, pre-pregnancy maternal medicine use, and pre-pregnancy healthcare utilisation.

*Comparator reference groups consisted of children that were not prenatally exposed to opioid analgesics, with three exceptions: (1) For discontinuers, the reference group included children prenatally exposed in the 90 days before the last menstrual period (LMP) but not during pregnancy; (2) for the sibling analysis, the reference group comprised siblings with no prenatal opioid exposure; and (3) for the OME comparison, low OME exposure was used as the reference group.


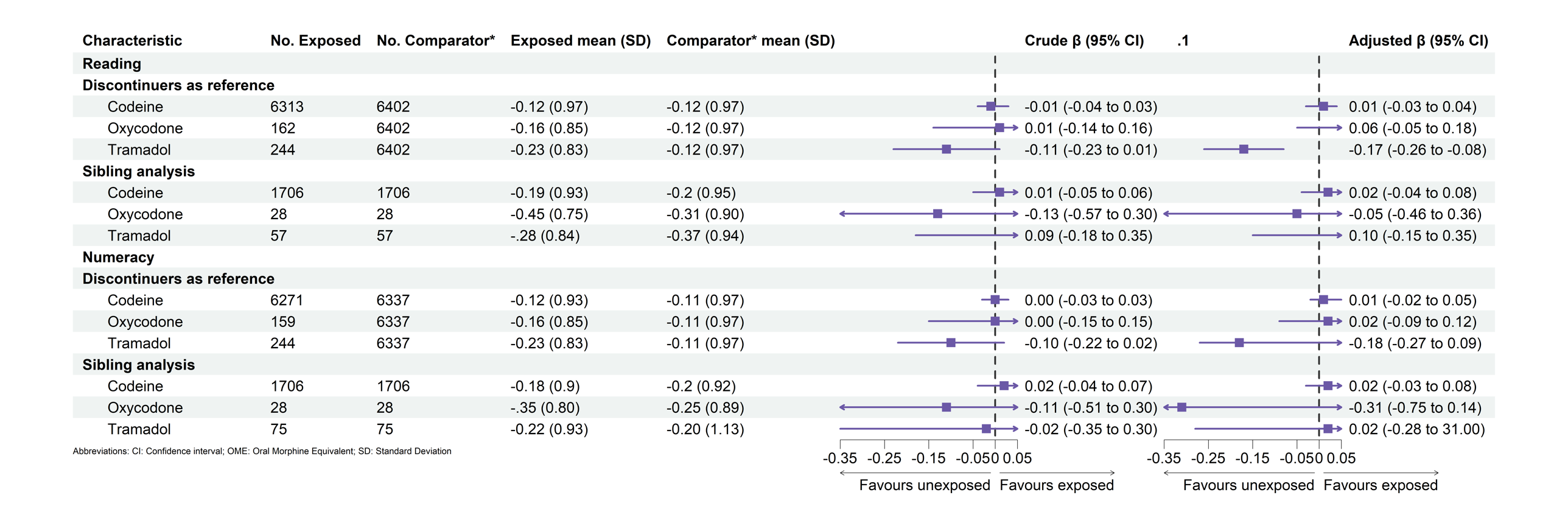


Figure S8: Differences in mean z-scores in reading and numeracy among third-grade children among children of discontinuers or unexposed siblings and among children prenatally exposed to opioid monotherapies. Crude and adjusted beta coefficients (β) with 95% confidence intervals (CI). Covariates included in the adjusted analyses included child’s sex, child’s year of birth, test year, language spoken at home, maternal age, born in western country, does not have a partner, social security benefits received, previous caesarean delivery, smoking during pregnancy, area-based socioeconomic disadvantage, area-based remoteness, multifetal gestation, parental highest educational attainment, parent occupation, maternal conditions, pre-pregnancy maternal medicine use, and pre-pregnancy healthcare utilisation.

*Comparator reference groups consisted of children that were not prenatally exposed to opioid analgesics, with three exceptions: (1) For discontinuers, the reference group included children prenatally exposed in the 90 days before the last menstrual period (LMP) but not during pregnancy; (2) for the sibling analysis, the reference group comprised of siblings with no prenatal opioid exposure; and (3) for the OME comparison, low OME exposure was used as the reference group.

Table S1: Exclusion criteria

| **Exclusion criteria** | **Timeframe** | **Data collection** | **ICD-10-AM, ATC codes, and other items** |
| --- | --- | --- | --- |
| Missing or implausible gestational age ^a^ (for example, birth weight < 1000 g while gestational age >38 weeks) | Ascertained at the time of birth | Perinatal records |  |
| NSW residence | Ascertained at the time of birth | Perinatal records |  |
| Treated or untreated opioid dependence | 12 months before LMP until childbirth | Hospital,  Mental health Ambulatory records | F11.1, F11.2, T40.0, T40.1, T40.2, T40.2^b^ |
|  |  | OAT records | Presence of authority for prescription of methadone or buprenorphine for opioid dependence treatment |
|  |  | PBS records | Naloxone V02AB15^c^ |

Abbreviations: NSW, New South Wales; OAT, opioid agonist therapy; OUD, opioid use disorder.
^a^ Records containing incompatible birthweight and gestational age combinations (such as birthweight <1000g at gestational age >38 weeks)(4) were flagged as data error and removed. ^b^ International Classification of Diseases, 10th Revision, Australian Modification (ICD-10-AM) codes
^c^ Anatomical Therapeutic Chemical Classification System Codes

Table S2: Prescription opioid analgesics included in the study and their corresponding Anatomical Therapeutic Chemical (ATC) Classification codes.

| **Analgesic Opioid** | **ATC code** |
| --- | --- |
| Codeine | R05DA04, N02AA |
| Codeine combination products | N02AA59, N02AJ06, N02AJ07, N02BA51, N02BE51 |
| Buprenorphine ^a^ | N02AE01 |
| Dextropropoxyphene ^b^ | N02AC04 |
| Fentanyl | N02AB03 |
| Hydromorphone | N02AA03 |
| Methadone ^a^ | N02AC |
| Morphine | N02AA01 |
| Oxycodone | N02AA05 |
| Pethidine (meperidine) ^c^ | N02AB02 |
| Tramadol | N02AX02 |

^a^ At the time of the study, buprenorphine and methadone within the PBS data were only indicated for pain; their use for opioid dependence was recorded in a separate dataset.
^b^ Removed from the PBS in 2011.
^c^ Removed from the PBS in 2007.

Table S3: List of covariates, relevant databases, ascertainment window and codes used for their identification.

| **Covariate** | **Data source** | **Ascertainment window** | | **Relevant codes^a^** |
| --- | --- | --- | --- | --- |
| **Child factors** |  |  | |  |
| Child’s sex  (Categorical: Male, Female, Indeterminate/Unknown) | Perinatal records | Recorded at the time of childbirth | |  |
| Child’s year of birth (Categorical: 2003, 2004, 2005, 2006, 2007, 2008, 2009, 2010, 2011, 2012) | Perinatal records | Recorded at the time of childbirth | |  |
| Test year  (Categorical: 2012, 2013, 2014, 2015, 2016, 2017, 2018, 2019) | NAPLAN records | First available third grade record. | |  |
| Language spoken at home  (Categorical: English, Non-English, Missing) | NAPLAN records | Recorded at initial NSW government school enrolment. | |  |
| **Maternal factors** | | | | |
| Maternal age at child’s birth  (Continuous: spline) | Perinatal records | Date of birth recorded at the time of childbirth.  Supplemented:  NSW Registry of Births, Deaths and Marriages, hospital records and emergency department records. | | Missing information was imputed using the median maternal age of 29 years (3/85,478 [0.0%] missing) |
| Born in Western Country   (Dichotomous) | Perinatal records | Recorded at the time of childbirth and supplemented with hospital records. | | Western countries included Australia, New Zealand, Europe, and North America. |
| Does not have a partner  (Dichotomous: does not have a partner/has a partner) | Hospital records | Recorded at the time of childbirth and supplemented with social security records. | | Missing information was imputed using the most common marital status, which was 'having a partner' (253/85,478 [0.3%] missing). |
| Social security benefits received | Social security records | Payment recorded upon receipt, whether single or recurring | |  |
| Parity   (Categorical: first born child, second born child, third born child, fourth born child or more) | Perinatal records | Recorded at the time of childbirth. | | Missing information was imputed using the most common parity status, which was ‘Second born child’ (37/85,478 [0.0%] missing). |
| Previous caesarean delivery  (Dichotomous: No/Yes) | Perinatal records | Recorded at the time of childbirth | | Missing information was imputed using the most common previous caesarean delivery status, which was ‘No’ (37/85,478 [0.2%] missing). |
| Smoking during pregnancy  (Dichotomous: No/Yes) | Perinatal records | Recorded at the time of childbirth. | | Recorded as smoking during the first half or second half of pregnancy. |
|  | Hospital records | Records from the year prior to LMP until the day before childbirth. | | Z72.0 |
| Quintiles of area-based socioeconomic disadvantage (IRSD)  (Categorical: 1(Most disadvantaged) /2/3/4/5 (Least disadvantaged) | Perinatal records | Recorded at the time of childbirth and supplemented with hospital records. | | Missing data were imputed using the median quintile value of 3 (10/85,478 [0.0%] missing). |
| Area-based remoteness (ARIA) (Categorical: Major cities/inner regional/outer regional/remote and very remote) | Perinatal records | Recorded at the time of childbirth and supplemented with hospital records. | | Missing information was imputed using the median quintile value, which was ‘Major Cities of Australia’ (41/85,478 [0.0%] missing). |
| Multifetal gestation  (Dichotomous: No/Yes) | Perinatal records | Recorded at the time of childbirth. | |  |
| Parental highest educational attainment  (Categorical: Bachelor’s degree or above, certificate or diploma, year 12 or equivalent, less than year 12, missing) | NAPLAN records | Recorded at initial NSW government school enrolment. | A separate category was created for those with missing information. | |
| Parent Occupation  (Categorical: group 1, group 2, group 3, group 4, no paid work in the previous 12 months, missing). | NAPLAN records | Recorded at initial NSW government school enrolment. | A separate category was created for those with missing information. | |
| **Maternal conditions** |  |  |  | |
| Anaemia and coagulation  (Dichotomous: No/Yes) | Hospital records | Records from the year prior to LMP until the day before childbirth. | | D50-D53, D55-D68 |
|  | Medicine dispensing records | Records from the year prior to LMP until the day before childbirth. | | Anticoagulants: B01AA03 - B01AB06, B01AE07, B01AF01, B01AF02, B01AX05    Antiplatelets: B01AC04 - B01AC07, B01AC12 - B01AC30, PBS items (05030R, 05035B, 05042J, 10111E, 10117L, 10129D, 10130E, 05751Q, 06456T, 11065J, 11082G, 11069N, 1109Q) |
| Cardiovascular disease  (Dichotomous: No/Yes) | Hospital records | Records from the year prior to LMP until the day before childbirth. | | Records from the year prior to trial entry until delivery: I05-I09, I34-I39, I50, I20, I25, I27, I28, Q20-Q25, O99.4, U82.1, U82.2    Records from the year prior to trial entry until trial entry: I00-I02, I21-I24, I26, I30-I33, I40-I43, I44-I49, I51-I52, I60-I64, G45.8, G45.9, I65, I66, I67.2, I70, I73, I74, I77, U82.1, U82.2 |
|  | Medicine dispensing records | Records from the year prior to LMP until the day before childbirth. | | Congestive heart failure: C01EB17, C09DX04, C03DA02 - C03DA99, C07AB07, C07AB12, C07AG02, [(C03CA01 - C03CC01) and (C09AA01- C09AX99 or C09CA01 - C09CX99)], PBS items (08732N, 08733P, 08734Q, 08735R) ^‡^    Ischaemic heart disease-hypertension: C07AA01 - C07AA06, C07AA08 - C07AB01, [C07AB02 if PBS item code is not (08732N, 08733P, 08734Q, 08735R) ^‡^], C07AB03, C07AG01, C08CA01 - C08DB01, C09BB02 - C09BB10, C09DB01 - C09DB04, C09DX01, C09DX03, C10BX03*    Ischaemic heart disease-angina: C01DA02-C01DA14, C01DX16, C08EX02 |
| Cancer diagnosis or treatment  (Dichotomous: No/Yes) | Hospital records | Records from the year prior to LMP until the day before cohort entry. | | C00-C97, Z51.1 |
|  | Medicine dispensing records | Records from the year prior to LMP until the day before cohort entry. | | L01AA01-L01AX04, L01BA01^a^, L01BA03- L01XX53, L02BA01^c^, L02BG03, L02BG04, L02BG06, L02BB01-L02BB04, L02BX01-L02BX03, L04AX02, L04AX04, L04AX06, L02AE02^d^, L02AE03^e^ |
| Chronic liver disease  (Dichotomous: No/Yes) | Hospital records | Records from the year prior to LMP until the day before childbirth. | | B18, K70.0 - K70.3, K70.9, K71.3 - K71.5, K71.7, K73, K74, K76.0, K76.2 - K76.4, K76.8, K76.9, Z94.4 |
| Chronic renal disease  (Dichotomous: No/Yes) | Hospital records | Records from the year prior to LMP until the day before childbirth. | | Records from the year prior to trial entry until delivery: N02-N08, N1-N12, N14-N16, N18-N19, N25-N28, Q60-Q63, N39.1, N39.2, T82.4, T86.1, Z49, Z94.0, Z99.2    Records from the year prior to trial entry until trial entry: N00, N01, N17 |
|  | Medicine dispensing records | Records from the year prior to LMP until the day before childbirth. | | A11CC04 (PBS authority code 5401), B03XA01 – B03XA08, V03AE02, V03AE03, V03AE05 |
| Drug and alcohol disorder  (Dichotomous: No/Yes) | Hospital records | Records from the year prior to LMP until the day before childbirth. | | F10-F19, Z50.2, Z50.3, Z72.1, Z72.2 |
|  | Medicine dispensing records | Records from the year prior to LMP until the day before childbirth. | | Alcohol dependency: N07BB01 - N07BB99  Smoking cessation pharmacotherapies:  N07BA01-N07BA02 |
| Epilepsy  (Dichotomous: No/Yes) | Hospital records | Records from the year prior to LMP until the day before childbirth. | | G40, F80.3 |
|  | Medicine dispensing records | Records from the year prior to LMP until the day before childbirth. | | N03AA01-N03AD51, N03AF01-N03AX11, N03AX13-N03AX30 |
| Pre-existing hypertension  (Dichotomous: No/Yes) | Hospital records | Records from the year prior to LMP until the day before childbirth. | | I10-I15, O10, U82.3 |
|  | Medicine dispensing records | Records from the year prior to LMP until the day before childbirth. | | C03AA01–C03BA11, C03DB01, C03DB99, C03EA01, C09BA02–C09BA09, C09DA01–C09DA08, C02AB01–C02AC05, C02DB02–C02DB99 (C03AA01–C03AX99 or C09CA01–C09CX99) |
|  | Perinatal data | Recorded at the time of childbirth. | |  |
| Pre-existing diabetes  (Dichotomous: No/Yes) | Hospital records | Records from the year prior to LMP until the day before childbirth. | | E10, E11, E13, E14, O24.0-O24.3 |
|  | Medicine dispensing records | Records from the year prior to LMP until the day before childbirth. | | A10A, A10BB – A10BK |
|  | Perinatal records | Recorded at the time of childbirth. | |  |
|  |  |  | |  |
| Mental health  (Dichotomous: No/Yes) | Hospital records | Records from the year prior to LMP until the day before childbirth. | | F32.0-F32.21, F32.8, F32.9, F33, F34, F38- F41, F48, O99.3, U79.3 |
|  | Medicine dispensing records | Records from the year prior to LMP until the day before childbirth. | | Anxiety: N05BA01 - N05BA12, N05BE01  Depression: N06AA01-N06AG02, N06AX03 - N06AX11, N06AX13 - N06AX18, N06AX21 - N06AX26 |
| Severe mental health  (Dichotomous: No/Yes) | Hospital records | Records from the year prior to LMP until the day before childbirth. | | F20-F25, F28-F31, F32.20, F32.3, F32.30, F32.31, F44, U79.2 |
|  | Medicine dispensing records | Records from the year prior to LMP until the day before childbirth. | | Bipolar: N05AN01  Psychotic illness: N05AA01 - N05AB02, N05AB06 - N05AL07, N05AX07 - N05AX13 |
| Obesity  (Dichotomous: No/Yes) | Hospital records | Records from the year prior to LMP until the day before childbirth. | | E66.0, E66.01, E66.09, E66.1, E66.2, E66.3, E66.8, E66.9, U78.1 |
| Respiratory disease  (asthma and chronic obstructive pulmonary disease [COPD])    (Dichotomous: No/Yes) | Hospital records | Records from the year prior to LMP until the day before childbirth. | | Records from the year prior to trial entry until delivery.  J40-J44 (COPD)    Records from the year prior to trial entry until trial entry: J45, J46, U83.3 (Asthma) |
|  | Medicine dispensing records | Records from the year prior to LMP until the day before childbirth. | | Records from the year prior to trial entry until delivery: R03AC18, R03AL03, R03AL04, R03AL05, R03AL06, R03BB04, R03BB05, R03BB06, R03BB07, PBS item codes 10018G, 10199T, 8432T, 8519J, 8750M (COPD)    Records from the year prior to trial entry until trial entry: R03AK, R03BA, R03DC (Asthma) |
| Thyroid disease  (Dichotomous: No/Yes) | Hospital records | Records from the year prior to LMP until the day before childbirth. | | E00-E07, E89.0 |
|  | Medicine dispensing records | Records from the year prior to LMP until the day before childbirth. | | Hyperthyroidism: H03BA02 - H03BB01    Hypothyroidism: H03AA01 - H03AA02 |
| Chronic viral hepatitis and HIV  (Dichotomous: No/Yes) | Hospital records | Records from the year prior to LMP until LMP. | | B18.2, B20-B24, B94.2, Z22.5 |
|  | Medicine dispensing records | Records from the year prior to LMP until LMP. | | J05AE01-J05AE12, J05AE14, J05AF01-J05AG05, J05AR01-J05AR99, J05AX07- J05AX09, J05AX12, J05AX14, J05AX15, J05AX65, |
| Rheumatic disease  (Dichotomous: No/Yes) | Hospital records | Records from the year prior to LMP until the day before childbirth. | | D89.1, M05, M06.1, M30.0, M30.1, M30.8, M31.3-M31.7, M32, M33.0-M33.9, M34, M35.0, M35.2, M35.3, M34, M94.1 |
|  | Medicine dispensing records | Records from the year prior to LMP until the day before childbirth. | | A07EC, L01BA (item codes, 01622J, 01623K, 2272N), L04AA, L04AX, M01C, P01BA, |
| Surgery  (Dichotomous: No/Yes) | Hospital records | Records from the year prior to LMP until LMP. | | Procedural block codes including 1-25, 27-43, 45-56, 58-86, 110-119, 121-129, 160-256, 300-333, 370-422, 520-570, 600-650, 653-657, 659-691, 693-748, 750-754, 758-768, 800-817, 850-884, 886-918, 924-942, 951-994, 996-998, 1000-1009, 1040-1082, 1084-1129, 1162-1203, 1240-1266, 1268-1299, 1360-1371, 1373-1379, 1381-1390, 1393-1406, 1408-1419, 1438-1474, 1478-1493, 1495-1524, 1528-1534, 1536-1548, 1552-1576, 1603-1607, 1630-1636, 1640-1718, 1740-1759. |
| Musculoskeletal pain  (Dichotomous: No/Yes) | Hospital records | Records from the year prior to LMP until LMP. | | M07, M13, M15.0, M15.1, M15.2, M15.4, M15.8, M15.9, M40.0, M40.2, M40.3, M40.4, M40.5, M41, M42, M43, M45, M46.0, M46.1, M46.2, M47, M48.0, M48.1, M48.2, M48.5, M48.8, M48.9, G95.0, G95.1 (Joint and spinal disease)  M79.7 (Fibromyalgia) |
| **Pre-pregnancy maternal medicine use** | | | | |
| Metformin  (Dichotomous: No/Yes) | Medicine dispensing records | Records from the year prior to LMP until LMP. | | A10BA |
| Non-opioid analgesics  (Dichotomous: No/Yes) | Medicine dispensing records | Records from the year prior to LMP until LMP. | | M01AB01 – M01AH06 (non-steroidal anti-inflammatory drugs [NSAIDS], N02BE01 (paracetamol), |
| Gabapentinoids  (Dichotomous: No/Yes) | Medicine dispensing records | Records from the year prior to LMP until LMP. | | N02BG (pregabalin), N02BG PBS items codes 04591P, 04592Q, 04593R, 04594T, 04595W (gabapentin) |
| Opioids  (Dichotomous: No/Yes) | Medicine dispensing records | Records from the year prior to LMP until 90 days before LMP. | | See codes in Table S2. |
| Psychotropics  (Dichotomous: No/Yes) | Medicine dispensing records | Records from the year prior to LMP until LMP. | | N03A, N05A, N05B, N05C, N06A, N02AX16, N05BA12, N05BA01, N05BA04, N05CD02, N0CD07. |
| Pregnancy Category D/X medicines (suspected teratogens, medicines with other adverse effects)  (Dichotomous: No/Yes) | Medicine dispensing records | Records during pregnancy until the day before childbirth. | | ATC codes listed in (5). |
| Systemic corticosteroids  (Dichotomous: No/Yes) | Medicine dispensing records | Records from the year prior to LMP until LMP. | | H02AB01-H02AB10 |
| **Pre-pregnancy maternal healthcare utilisation** | | | | |
| Number of hospital admissions  (Categorical: 0, 1, ≥2) | Hospital records | Records from the year prior to LMP until LMP. | |  |
| Number of ED presentations  (Categorical: 0, 1, ≥2) | Emergency department presentation records | Records from the year prior to LMP until LMP. | |  |
| Number of GP visits  (Categorical: 0, 1, ≥2) | MBS records  . | Records from the year prior to LMP until LMP | | MBS category: item numbers  A1 GP: 3, 23, 36, 44 (in surgery visits), 4, 24, 37, 47 (Home/institution/hospital visits).  A5 Prolonged: 160.  A11 After hours: 585, 599.  A22 GP after-hours attendances to which no other item applies: 5000, 5003, 5010, 5020, 5023, 5028, 5040, 5043, 5049, 5060, 5063, 5067.  A30: Medical practitioner video conferencing consultation: 2100, 2122, 2126, 2137, 2143, 2147, 2195, 2199, 2125, 2138, 2179, 2220 |

Abbreviations: Emergency Department, ED; General Practitioner, GP; Human Immunodeficiency Virus, HIV; Last Menstrual Period, LMP; Medicare Benefits Schedule, MBS; National Assessment Program - Literacy and Numeracy, NAPLAN
^a^ Relevant classification systems: ATC (Anatomical Therapeutic Chemical) for medicines, ICD-10-AM (International Classification of Diseases, 10th Edition, Australian Modification) for hospital diagnoses, and ACHI (Australian Classification of Health Interventions) for hospital procedures.

Table S4: List of birth outcomes, data sources and codes used for their identification

| **Birth outcomes** | **Data Source & definition** | **ICD-10-AM diagnosis Codes** | **ACHI procedure Codes** | **Citation or validation results** |
| --- | --- | --- | --- | --- |
| Placental abruption | Hospital records when gestational age was ≥20 weeks. | O45 |  | Validated during a women’s delivery admission: PPV: 100% (95% CI: 27.6% - 100%) (6). |
| Preterm premature rupture of membranes | Hospital records when gestational age at birth was <37 weeks. | O42 |  |  |
| Preterm birth | Preterm birth is characterized by childbirth occurring before 37 completed weeks, either as a result of spontaneous onset of labour or medical intervention and is determined based on the gestational age at delivery and the onset of labour (recorded in the perinatal record). |  |  |  |
|  |  |  |  |  |
| Severe neonatal morbidity complications | Obtained from perinatal records, baby’s birth admission or in any hospital transfer admission that occurred prior to the first discharge home, and mortality records. | Gestational age<32 weeks, birthweight<1500g, perinatal death, respiratory distress syndrome (P22.0), Seizure (P90, R56); Intraventricular haemorrhage (grades 2, 3 and 4) (P52.1, P52.2); Cerebral infarction (I63); Periventricular leukomalacia (P91.2); Birth trauma (intracranial haemorrhage, paralysis due to brachial plexus injury, skull, or long bone fracture) (P10.0 – P10.3, P13.0, P13.2, P13.3, P14.0, P14.1); Hypoxic ischemic encephalopathy (P91.5, P91.81, P91.6); Necrotising enterocolitis (P77); Broncho-pulmonary dysplasia (P27.1); Sepsis/septicaemia (streptococcus, staphylococcus, E. coli, unspecified Gram-negative) (P36, A40, A41.5, A41.9, B95.1, B96.2); Pneumonia (P23, J12 to J18); Other respiratory: primary atelectasis, respiratory failure (P28.0, P28.5). | Resuscitation (92052, 92053, 92042–00, 90225); Ventilatory support (mechanical ventilation and/or CPAP) (13882, 13857–00, 13879–00, 22007, 90179, 92038, 92039); Central venous or arterial catheter (38206, 13303–00, 34524–00, 34530–01, 13300–00, 13300–02, 13319–00, 13815); Transfusion of blood or blood products (13706–01 to 04, 92206–00, 13306–00); Pneumothorax requiring an intercostal catheter (38409–00); Any body cavity surgical procedure (30373, 30375, 30378–00, 30562, 30564 to 30566, 30571, 30601, 30615–00, 30617–00, 32123–00, 36516, 36537, 36564, 36579, 38403–00, 38600–00, all codes start with 387, 39015, 39640–00, 40003, 40100–00, 40103–00, 41883, 43801–00, 43807–00, 43816–02, 43837, 43843, 43852–00, 43864, 43867, 43870–00, 43873, 43876, 43900–00, 43915–00, 43930–00, 43945–00, 43963–00, 43978, 90180, 90224–00); Any intravenous fluids (96199). | Validated (7). |
| Apgar score (<7) at 5 minutes | Obtained from the perinatal records.  Clinical indicators of a baby’s condition shortly after birth obtained from the perinatal record. A score of less than 7 indicates complications for the baby. |  |  |  |
| Small for gestational age | Obtained from the perinatal records:  SGA was defined as birthweight of less than the tenth percentile specific for sex- and gestational-age according to Australian national birthweight percentiles in singleton pregnancies.(4) |  |  |  |
| Neonatal abstinence syndrome | Diagnosis was recorded in women’s childbirth admission, baby’s birth admission or any infant hospital admission within 28 days of childbirth. | P96.1 |  |  |

Abbreviations: ACHI, Australian Classification of Health Interventions; ICD-10-AM, International Classification of Diseases, 10th Edition, Australian Modification; PPV, positive predictive value.

Table S5: Comparison of children characteristics of those with test scores and those classified as lost to follow-up.

| **Characteristics** | **Children with test scores**  **(n=70,882)**  No. (%) | **Children lost to follow-up**  **(n=9,972)**  No. (%) | **Absolute standardized differences** | |
| --- | --- | --- | --- | --- |
| ***Child factors*** |  |  |  | |
| Child’s sex |  |  |  | |
| Male | 35,250 (49.7) | 5,092 (51.1) | 0.03 | |
| Female | 35,623 (50.3) | 4,873 (48.9) | 0.03 | |
| Indeterminate/Unknown | 9 (0.0) | 7 (0.1) | 0.03 | |
| Child’s year of birth |  |  |  | |
| 2003 | 1,680 (2.4) | 257 (2.6) | 0.01 | |
| 2004 | 6,706 (9.5) | 986 (9.9) | 0.01 | |
| 2005 | 8,015 (11.3) | 1,177 (11.8) | 0.02 | |
| 2006 | 8,658 (12.2) | 1,180 (11.8) | 0.01 | |
| 2007 | 9,399 (13.3) | 1,311 (13.1) | 0.00 | |
| 2008 | 10,034 (14.2) | 1,501 (15.1) | 0.03 | |
| 2009 | 10,574 (14.9) | 1,523 (15.3) | 0.01 | |
| 2010 | 11,918 (16.8) | 2,037 (20.4) | 0.09 | |
| 2011^a^ | 3,898 (5.5) | 0 (0.0) | 0.46 | |
| ***Maternal factors*** |  |  |  | |
| Maternal age at child’s birth (years), Mean (SD) | 29.4 (5.9) | 28.7 (5.8) | 0.13 | |
| <25 | 16,507 (23.3) | 2,742 (27.5) | 0.10 | |
| 25 to 34 | 39,175 (55.3) | 5,428 (54.4) | 0.02 | |
| 35+ | 15,200 (21.4) | 1,802 (18.1) | 0.09 | |
| Born in a Western Country | 15,054 (21.2) | 1,516 (15.2) | 0.16 | |
| Does not have a partner | 24,970 (35.2) | 3,706 (37.2) | 0.04 | |
| Social security benefits received^b^ | 16,108 (22.7) | 2,025 (20.3) | 0.06 | |
| Parity |  |  |  | |
| First born child | 5,326 (7.5) | 836 (8.4) | 0.03 | |
| Second born child | 24,390 (34.4) | 3,042 (30.5) | 0.08 | |
| Third born child | 20,389 (28.8) | 2,621 (26.3) | 0.06 | |
| Fourth born child or more | 20,777 (29.3) | 3,473 (34.8) | 0.12 | |
| Previous caesarean delivery | 11,501 (16.2) | 1,581 (15.9) | 0.01 | |
| Smoking during pregnancy | 6,019 (8.5) | 1,037 (10.4) | 0.07 | |
| Quintiles of area-based residential socioeconomic disadvantage (IRSD) |  |  |  | |
| Most disadvantaged | 31,540 (44.5) | 4,453 (44.7) | 0.00 | |
| 2 | 16,748 (23.6) | 2,530 (25.4) | 0.04 | |
| 3 | 13,029 (18.4) | 1,692 (17.0) | 0.04 | |
| 4 | 5,388 (7.6) | 716 (7.2) | 0.02 | |
| Least disadvantaged | 4,177 (5.9) | 581 (5.8) | 0.00 | |
| Area-based residential remoteness (ARIA) |  |  |  | |
| Major Cities of Australia | 47,985 (67.7) | 5,904 (59.2) | 0.18 | |
| Inner Regional Australia | 16,056 (22.7) | 2,724 (27.3) | 0.11 | |
| Outer Regional Australia | 6,169 (8.7) | 1,232 (12.4) | 0.12 | |
| Remote & Very Remote Australia | 672 (0.9) | 112 (1.1) | 0.02 | |
| Multifetal gestation | 2,039 (2.9) | 319 (3.2) | 0.02 | |
| ***Maternal conditions (year prior to LMP until the day before childbirth)*** | | | |  |
| Anaemia and coagulation | 1,216 (1.7) | 169 (1.7) | 0.00 | |
| Cardiovascular disease | 1,699 (2.4) | 203 (2.0) | 0.03 | |
| Cancer diagnosis or treatment | ≤5 (0.0) | ≤5 (0.0) | 0.00 | |
| Chronic liver disease | 26 (0.0) | ≤5 (0.0) | 0.00 | |
| Chronic renal disease | 48 (0.1) | 8 (0.1) | 0.01 | |
| Drug and alcohol disorder | 770 (1.1) | 120 (1.2) | 0.01 | |
| Epilepsy | 76 (0.1) | 14 (0.1) | 0.01 | |
| Pre-existing hypertension | 342 (0.5) | 54 (0.5) | 0.01 | |
| Pre-existing diabetes | 326 (0.5) | 35 (0.4) | 0.02 | |
| Mental health | 10,923 (15.4) | 1,701 (17.1) | 0.05 | |
| Severe mental health | 964 (1.4) | 176 (1.8) | 0.03 | |
| Obesity | 147 (0.2) | 18 (0.2) | 0.01 | |
| Respiratory disease | 8 (0.0) | ≤5 (0.0) | 0.00 | |
| Thyroid disease | 1,178 (1.7) | 126 (1.3) | 0.03 | |
| Chronic viral hepatitis and HIV | 251 (0.4) | 37 (0.4) | 0.00 | |
| Rheumatic disease | 290 (0.4) | 33 (0.3) | 0.01 | |
| Surgery^c^ | 7 (0.0) | ≤5 (0.0) | ≤0.01 | |
| Musculoskeletal pain^c^ | 32 (0.0) | ≤5 (0.0) | ≤0.02 | |
| ***Pre-pregnancy maternal medicine use (year prior to LMP)*** | | | | |
| Metformin | 890 (1.3) | 93 (0.9) | 0.03 | |
| Non-opioid analgesics | 11,123 (15.7) | 1,297 (13.0) | 0.08 | |
| Gabapentinoids | 26 (0.0) | ≤5 (0.0) | 0.00 | |
| Opioids^d^ | 8,896 (12.6) | 1,310 (13.1) | 0.02 | |
| Psychotropics | 2,734 (3.9) | 474 (4.8) | 0.04 | |
| Pregnancy category D/X medicines^e^ | 2,565 (3.6) | 296 (3.0) | 0.04 | |
| Systemic corticosteroids | 3,320 (4.7) | 415 (4.2) | 0.03 | |
| \| ***Pre-pregnancy maternal healthcare utilisation (year prior to LMP)*** \| \| --- \| | | | | |
| Number of hospital admissions |  |  |  | |
| 0 | 49,574 (69.9) | 6,749 (67.7) | 0.05 | |
| 1 | 15,348 (21.7) | 2,237 (22.4) | 0.02 | |
| ≥2 | 5,960 (8.4) | 986 (9.9) | 0.05 | |
| Number of ED presentations |  |  |  | |
| 0 | 56,671 (80.0) | 7,910 (79.3) | 0.02 | |
| 1 | 8,586 (12.1) | 1,175 (11.8) | 0.01 | |
| ≥2 | 5,625 (7.9) | 887 (8.9) | 0.04 | |
| Number of GP visits |  |  |  | |
| 0 | 35,695 (50.4) | 5,077 (50.9) | 0.01 | |
| 1 | 17,750 (25.0) | 2,438 (24.4) | 0.01 | |
| ≥2 | 17,437 (24.6) | 2,457 (24.6) | 0.00 | |

Abbreviations: ED, Emergency Department; GP, General practitioner; SD: Standard deviation.
^a^ Differences in the last year of childbirth are likely attributable to our classification criteria, where children younger than nine years old as of the final test date (May 31, 2019) were categorized as too young for testing
^b^ Social security benefits refer to government-administered financial assistance provided to eligible individuals.
^c^ Ascertained in the year prior to LMP until LMP.
^d^ Ascertained in the year prior to LMP until 90 days before LMP.
^e^ Ascertained during pregnancy.

Table S6: Maternal and child characteristics by exposure to specific opioid analgesics during pregnancy, for children of concessional beneficiaries between January 01, 2003, to March 26, 2011.

| **Characteristics** | **Unexposed to opioids**  **(n=63,776)** No. (%) | **Exposed** | | | | | |
| --- | --- | --- | --- | --- | --- | --- | --- |
|  |  | **Codeine (n=6,375)** No. (%) | **Absolute SD (%) relative to the unexposed group**^a^ | **Oxycodone (n=163)** No. (%) | **Absolute SD (%)  relative to the unexposed group**^a^ | **Tramadol (n=252)** No. (%) | **Absolute SD (%) relative to the unexposed group**^a^ |
| ***Child factors*** |  |  |  |  |  |  |  |
| Child’s sex |  |  |  |  |  |  |  |
| Male | 31,716 (49.7) | 3163 (49.6) | 0.00 | 87 (53.4) | 0.07 | 131 (52.0) | 0.04 |
| Female | 32,042 (50.2) | ^a^ | 0.00 | 76 (46.6) | 0.07 | 121 (48.0) | 0.04 |
| Indeterminate/Unknown | 8 (0.0) | ≤5 | 0.00 | 0 | 1.32 | 0 | 1.36 |
| Child’s year of birth |  |  |  |  |  |  |  |
| 2003 | 1,502 (2.4) | 168 (2.6) | 0.02 | ≤5 | ≥0.10 | ≤5 | ≤0.10 |
| 2004 | 6,068 (9.5) | 577 (9.1) | 0.02 | ^a^ | ≥0.10 | ^a^ | ≤0.10 |
| 2005 | 7,163 (11.2) | 777 (12.2) | 0.03 | 8 (4.9) | 0.23 | 29 (11.5) | 0.01 |
| 2006 | 7,815 (12.3) | 742 (11.6) | 0.02 | 15 (9.2) | 0.10 | 42 (16.7) | 0.13 |
| 2007 | 8,461 (13.3) | 850 (13.3) | 0.00 | 11 (6.7) | 0.22 | 30 (11.9) | 0.04 |
| 2008 | 8,989 (14.1) | 952 (14.9) | 0.02 | 20 (12.3) | 0.05 | 30 (11.9) | 0.07 |
| 2009 | 9,535 (15.0) | 930 (14.6) | 0.02 | 29 (17.8) | 0.08 | 33 (13.1) | 0.05 |
| 2010 | 10,734 (16.8) | 1037 (16.3) | 0.01 | 56 (34.4) | 0.41 | 38 (15.1) | 0.05 |
| 2011 | 3,499 (5.5) | 342 (5.4) | 0.01 | 17 (10.4) | 0.18 | 16 (6.3) | 0.04 |
| Test year |  |  |  |  |  |  |  |
| 2011 | ≤5 | 0 |  | 0 |  | 0 |  |
| 2012 | ^a^ | 370 (5.8) | 0.02 | ≤5 | ≥0.10 | ≤5 | ≤0.10 |
| 2013 | 6447 (10.1) | 622 (9.8) | 0.01 | ^a^ | ≥0.10 | ^a^ | ≤0.10 |
| 2014 | 7305 (11.5) | 765 (12.0) | 0.02 | 8 (4.9) | 0.24 | 31 (12.3) | 0.03 |
| 2015 | 8040 (12.6) | 772 (12.1) | 0.02 | 17 (10.4) | 0.07 | 41 (16.3) | 0.10 |
| 2016 | 8620 (13.5) | 852 (13.4) | 0.00 | 10 (6.1) | 0.25 | 28 (11.1) | 0.07 |
| 2017 | 9111 (14.3) | 970 (15.2) | 0.03 | 23 (14.1) | 0.01 | 32 (12.7) | 0.05 |
| 2018 | 9778 (15.3) | 948 (14.9) | 0.01 | 38 (23.3) | 0.20 | 36 (14.3) | 0.03 |
| 2019 | 11099 (17.4) | 1076 (16.9) | 0.01 | 58 (35.6) | 0.42 | 40 (15.9) | 0.04 |
|  |  |  |  |  |  |  |  |
| Language spoken at home |  |  |  |  |  |  |  |
| English | 45,879 (71.9) | 4641 (72.8) | 0.02 | 139 (85.3) | 0.33 | 184 (73.0) | 0.02 |
| Non-English language | 17,005 (26.7) | 1650 (25.9) | 0.02 | ^a^ | ≥0.10 | ^a^ | <0.10 |
| Missing | 882 (1.4) | 84 (1.3) | 0.01 | ≤5 | ≥0.10 | ≤5 | ≥0.10 |
| Infant age at test (months), mean (standard deviation) | 102.5 (4.5) | 102.4 (4.5) | 0.03 | 103.0 (5.1) | 0.10 | 102.4 (4.7) | 0.02 |
| ***Maternal factors*** |  |  |  |  |  |  |  |
| Maternal age at child’s birth (years) Mean (standard deviation) | 29.5 (5.9) | 29.0 (5.8) | 0.80 | 30.0 (5.8) | 0.09 | 30.1 (6.1) | 0.11 |
| <25 | 14,766 (23.2) | 1,606 (25.2) | 0.05 | 30 (18.4) | 0.12 | 52 (20.6) | 0.06 |
| 25 to 34 | 35,226 (55.2) | 3,524 (55.3) | 0.00 | 96 (58.9) | 0.07 | 135 (53.6) | 0.03 |
| 35+ | 13,774 (21.6) | 1,245 (19.5) | 0.05 | 37 (22.7) | 0.03 | 65 (25.8) | 0.10 |
| Born in a Western Country | 13,666 (21.4) | 1,303 (20.4) | 0.02 | 13 (8.0) | 0.39 | 42 (16.7) | 0.12 |
| Does not have a partner | 21,910 (34.4) | 2,737 (42.9) | 0.18 | 67 (41.1) | 0.14 | 111 (44.0) | 0.20 |
| Social security benefits received^b^ | 14,405 (22.6) | 1493 (23.4) | 0.02 | 73 (44.8) | 0.48 | 56 (22.2) | 0.01 |
| Parity |  |  |  |  |  |  |  |
| First born child | 4,777 (7.5) | 499 (7.8) | 0.01 | 11 (6.7) | 0.03 | 19 (7.5) | 0.00 |
| Second born child | 22,090 (34.6) | 2087 (32.7) | 0.04 | 49 (30.1) | 0.10 | 70 (27.8) | 0.15 |
| Third born child | 18,458 (28.9) | 1733 (27.2) | 0.04 | 44 (27.0) | 0.04 | 72 (28.6) | 0.01 |
| Fourth born child or more | 18,441 (28.9) | 2056 (32.3) | 0.07 | 59 (36.2) | 0.16 | 91 (36.1) | 0.15 |
| Previous caesarean delivery | 10,289 (16.1) | 1050 (16.5) | 0.01 | 33 (20.2) | 0.11 | 54 (21.4) | 0.14 |
| Smoking during pregnancy | 4,579 (7.2) | 1221 (19.2) | 0.36 | 51 (31.3) | 0.64 | 66 (26.2) | 0.53 |
| Quintiles of area-based socioeconomic disadvantage (IRSD) |  |  |  |  |  |  |  |
| Most disadvantaged | 28,141 (44.1) | 3066 (48.1) | 0.08 | 71 (43.6) | 0.01 | 128 (50.8) | 0.13 |
| 2 | 15,009 (23.5) | 1564 (24.5) | 0.02 | 44 (27.0) | 0.08 | 50 (19.8) | 0.09 |
| 3 | 11,760 (18.4) | 1124 (17.6) | 0.02 | 32 (19.6) | 0.03 | 44 (17.5) | 0.03 |
| 4 | 4,948 (7.8) | 389 (6.1) | 0.07 | 10 (6.1) | 0.06 | 17 (6.7) | 0.04 |
| Least disadvantaged | 3,908 (6.1) | 232 (3.6) | 0.12 | 6 (3.7) | 0.11 | 13 (5.2) | 0.04 |
| Area-based residential remoteness (ARIA) |  |  |  |  |  |  |  |
| Major Cities of Australia | 43,097 (67.6) | 4428 (69.5) | 0.04 | 77 (47.2) | 0.42 | 176 (69.8) | 0.05 |
| Inner Regional Australia | 14,479 (22.7) | 1376 (21.6) | 0.03 | 69 (42.3) | 0.43 | 49 (19.4) | 0.08 |
| Outer Regional Australia | 5,571 (8.7) | 522 (8.2) | 0.02 | ^a^ | ≤0.10 | ^a^ | ≤0.10 |
| Remote & Very Remote Australia | 619 (1.0) | 49 (0.8) | 0.02 | ≤5 | ≤0.10 | ≤5 | ≤0.10 |
| Multifetal gestation | 1,796 (2.8) | 220 (3.5) | 0.04 | ≤5 | ≤0.10 | 8 (3.2) | 0.02 |
| Parental^c^ highest educational attainment |  |  |  |  |  |  |  |
| Bachelor’s degree or above | 7,772 (12.2) | 473 (7.4) | 0.16 | 17 (10.4) | 5.6 | 18 (7.1) | 0.17 |
| Certificate or Diploma | 30,929 (48.5) | 3044 (47.7) | 0.02 | 76 (46.6) | 0.04 | 111 (44.0) | 0.09 |
| Year 12 or equivalent | 6,194 (9.7) | 619 (9.7) | 0.00 | 14 (8.6) | 0.04 | 32 (12.7) | 0.10 |
| <Year 12 | 15,954 (25.0) | 1951 (30.6) | 0.13 | 45 (27.6) | 0.06 | 80 (31.7) | 0.15 |
| Missing | 2,917 (4.6) | 288 (4.5) | 0.00 | 11 (6.7) | 0.09 | 11 (4.4) | 0.01 |
| Parent Occupation^d^ |  |  |  |  |  |  |  |
| Group 1 | 4,004 (6.3) | 264 (4.1) | 0.10 | 6 (3.7) | 0.12 | ≤5 | ≥0.10 |
| Group 2 | 7,966 (12.5) | 536 (8.4) | 0.13 | 17 (10.4) | 0.07 | * | ≥0.10 |
| Group 3 | 13,292 (20.8) | 1225 (19.2) | 0.04 | 37 (22.7) | 0.05 | 43 (17.1) | 0.10 |
| Group 4 | 16,172 (25.4) | 1703 (26.7) | 0.03 | 36 (22.1) | 0.08 | 54 (21.4) | 0.09 |
| No paid work in the previous 12 months | 16,269 (25.5) | 1985 (31.1) | 0.13 | 51 (31.3) | 0.13 | 89 (35.3) | 0.21 |
| Missing | 6,063 (9.5) | 662 (10.4) | 0.03 | 16 (9.8) | 0.01 | 34 (13.5) | 0.13 |
| ***Maternal conditions (year prior to LMP until the day before childbirth)*** | | | | | | | |
| Anaemia and coagulation | 989 (1.6) | 191 (3.0) | 0.10 | 13 (8.0) | 0.31 | ≤5 | ≤0.10 |
| Cardiovascular disease | 1,345 (2.1) | 307 (4.8) | 0.15 | 6 (3.7) | 0.09 | ≤5 | ≥0.10 |
| Cancer diagnosis or treatment | ≤5 | ≤5 | ≤0.10 | 0 | ≥0.10 | 0 | ≥0.10 |
| Chronic liver disease | 24 (0.0) | ≤5 | 0.00 | 0 | 0.27 | 0 | 0.33 |
| Chronic renal disease | 33 (0.1) | 12 (0.2) | 0.04 | ≤5 | ≤0.10 | 0 | 0.32 |
| Drug and alcohol disorder | 615 (1.0) | 139 (2.2) | 0.10 | 6 (3.7) | 0.18 | ≤5 | ≤0.10 |
| Epilepsy | 64 (0.1) | 10 (0.2) | 0.02 | ≤5 | ≤0.10 | ≤5 | ≤0.10 |
| Pre-existing hypertension | 286 (0.4) | 30 (0.5) | 0.01 | ≤5 | ≤0.10 | ≤5 | ≤0.10 |
| Pre-existing diabetes | 301 (0.5) | 33 (0.5) | 0.00 | 0 | 0.30 | ≤5 | ≤0.10 |
| Mental health | 8,859 (13.9) | 1722 (27.0) | 0.33 | 60 (36.8) | 0.55 | 101 (40.1) | 0.62 |
| Severe mental health | 775 (1.2) | 158 (2.5) | 0.09 | 9 (5.5) | 0.24 | ≤5 | 0.00 |
| Obesity | 124 (0.2) | 17 (0.3) | 0.02 | 0 | 0.23 | ≤5 | ≤0.10 |
| Respiratory disease | 7 (0.0) | 0 | 0.07 | 0 | 0.25 | ≤5 | ≤0.10 |
| Thyroid disease | 1,039 (1.6) | 118 (1.9) | 0.02 | 6 (3.7) | 0.13 | ≤5 | 0.00 |
| Chronic viral hepatitis and HIV | 197 (0.3) | 45 (0.7) | 0.06 | ≤5 | ≥0.10 | ≤5 | ≥0.10 |
| Rheumatic disease | 235 (0.4) | 44 (0.7) | 0.04 | ≤5 | ≤0.10 | ≤5 | 0.00 |
| Surgery^e^ | ≤5 | ≤5 | ≤0.10 | ≤5 | ≥0.10 | ≤5 | ≤0.10 |
| Musculoskeletal pain^e^ | 20 (0.0) | 10 (0.2) | 0.04 | ≤5 | ≥0.10 | 0 | ≥0.10 |
| ***Pre-pregnancy maternal medicine use (year prior to LMP)*** | | | | | | | |
| Metformin | 742 (1.2) | 127 (2.0) | 0.07 | ≤5 | ≥0.10 | ≤5 | ≤0.10 |
| Non-opioid analgesics | 8,944 (14.0) | 1860 (29.2) | 0.37 | 51 (31.3) | 0.42 | 109 (43.3) | 0.68 |
| Gabapentinoids | 15 (0.0) | 10 (0.2) | 0.04 | 0 | 0.95 | 0 | 1.23 |
| Opioids^f^ | 6,445 (10.1) | 2040 (32.0) | 0.56 | 77 (47.2) | 0.90 | 121 (48.0) | 0.92 |
| Psychotropics | 1,978 (3.1) | 608 (9.5) | 0.27 | 32 (19.6) | 0.54 | 47 (18.7) | 0.52 |
| Pregnancy category D/X medicines^g^ | 2,110 (3.3) | 394 (6.2) | 0.14 | 7 (4.3) | 0.05 | 25 (9.9) | 0.27 |
| Systemic corticosteroids | 2,653 (4.2) | 580 (9.1) | 0.19 | 14 (8.6) | 0.18 | 19 (7.5) | 0.14 |
| ***Pre-pregnancy maternal healthcare utilisation (year prior to LMP)*** | | | | | | | |
| Number of hospital admissions |  |  |  |  |  |  |  |
| 0 | 45,092 (70.7) | 4078 (64.0) | 0.14 | 87 (53.4) | 0.36 | 152 (60.3) | 0.22 |
| 1 | 13,673 (21.4) | 1484 (23.3) | 0.04 | 40 (24.5) | 0.07 | 66 (26.2) | 0.11 |
| ≥2 | 5,001 (7.8) | 813 (12.8) | 0.16 | 36 (22.1) | 0.41 | 34 (13.5) | 0.18 |
| Number of ED presentations |  |  |  |  |  |  |  |
| 0 | 51,618 (80.9) | 4593 (72.0) | 0.21 | 90 (55.2) | 0.57 | 170 (67.5) | 0.31 |
| 1 | 7,523 (11.8) | 952 (14.9) | 0.09 | 29 (17.8) | 0.17 | 39 (15.5) | 0.11 |
| ≥2 | 4,625 (7.3) | 830 (13.0) | 0.19 | 44 (27.0) | 0.54 | 43 (17.1) | 0.30 |
| Number of GP visits |  |  |  |  |  |  |  |
| 0 | 32,917 (51.6) | 2530 (39.7) | 0.24 | 74 (45.4) | 0.12 | 83 (32.9) | 0.39 |
| 1 | 15,988 (25.1) | 1608 (25.2) | 0.00 | 26 (16.0) | 0.23 | 62 (24.6) | 0.01 |
| ≥2 | 14,861 (23.3) | 2237 (35.1) | 0.26 | 63 (38.7) | 0.34 | 107 (42.5) | 0.42 |

Abbreviations: ED, Emergency Department; GP, General practitioners; HIV, Human Immunodeficiency Virus; SD, Standardised difference.
^a^ Suppressed to prevent calculations of cells with small count. Absolute standardised differences were suppressed and only reported as being above or below the meaningful difference threshold of 0.1.
^b^ Social security benefits refer to government-administered financial assistance provided to eligible individuals.
^c^ Parental characteristics, collected at initial NSW government school enrolment (typically before third grade), included education level and occupation of mother and second parent (if available).
^d^ Occupation group is completed by parents of children applying to enrol in a NSW government school for the first time. Examples of professionals listed in each group include:
Group 1: elected officials and senior managers.
Group 2: business managers and associate professors.
Group 3: tradespeople and advanced/intermediate clerical staff.
Group 4: machine operators, assistances, sales and service staff.
^e^Ascertained in the year prior to LMP until LMP.
^f^Ascertained in the year prior to LMP until 90 days before LMP.
^g^Ascertained during pregnancy.

Table S7: Crude and standardised proportions of birth outcomes per 100 infants with 95% confidence intervals among those with prenatal opioid exposure and those unexposed.

| **Birth outcomes** | **Exposure** | **Number of children with birth outcome^a^** | **Crude proportion per 100 children**  **(95% CI)** | **Standardised^b^ proportion per 100 children (95% CI)** |
| --- | --- | --- | --- | --- |
| Low Apgar score (<7) at 5 minutes | Unexposed | 1118 | 1.8 (1.7 – 1.9) | 2.0 (1.8 - 2.1) |
|  | Exposed | 130 |  | 1.8 (1.5 – 2.2) |
| Neonatal abstinence syndrome | Unexposed | 300 | 0.5 (0.4 – 0.5) | 0.8 (0. 7 – 1.0) |
|  | Exposed | 66 |  | 0.9 (0.7 – 1.2) |
| Placental abruption | Unexposed | 345 | 0.5 (0.5 – 0.6) | 0.8 (0.6 – 0.9) |
|  | Exposed | 70 |  | 1.0 (0.8 – 1.2) |
| Preterm birth | Unexposed | 4791 | 7.5 (7.3 – 7.7) | 9.4 (8.9 – 9.9) |
|  | Exposed | 652 |  | 9.2 (8.4 – 9.9) |
| Preterm premature rupture of membranes | Unexposed | 1230 | 1.9 (1.8 – 2.0) | 2.6 (2.3 – 2.9) |
|  | Exposed | 156 |  | 2.2 (1.8 – 2.6) |
|  |  |  |  |  |
| Severe neonatal morbidity composite | Unexposed | 2171 | 3.4 (3.3 – 3.6) | 4.3 (3.9 – 4.6) |
|  | Exposed | 301 |  | 4.2 (3.7 – 4.8) |
| Small for gestational age | Unexposed | 5707 | 8.9 (8.7 – 9.2) | 10.2 (9.8 – 10.7) |
|  | Exposed | 734 |  | 10.3 (9.6 – 11.1) |

^a^ Among children with test scores. Total: Unexposed: 63,766; Exposed, 7,166
^b^ Standardised on all covariates except test year and child sex.

Table S8: Comparison of characteristics of children of eligible concessional beneficiaries (study population) and those born to women that did not meet the continous concessional beneficiary status.

| **Characteristics** | **Non-continuous concessional**  **beneficiary**  **(n=653,660)**  No. (%) | **Eligible concessional beneficiary**  **(n=85,478)**  No. (%) | **Absolute standardized differences** |
| --- | --- | --- | --- |
| ***Child factors*** |  |  |  |
| Child’s sex |  |  |  |
| Male | 336,074 (51.4) | 49,740 (51.0) | 0.00 |
| Female | 317,261 (48.5) | 47,795 (49.0) | 0.00 |
| Indeterminate/Unknown | 325 (0.0) | 22 (0.0) | 0.00 |
| Child’s year of birth |  |  |  |
| 2003 | 18,443 (2.8) | 2,061 (2.4) | 0.03 |
| 2004 | 75,260 (11.5) | 8,146 (9.5) | 0.07 |
| 2005 | 78,459 (12.0) | 9,734 (11.4) | 0.02 |
| 2006 | 79,883 (12.2) | 10,396 (12.2) | 0.00 |
| 2007 | 82,209 (12.6) | 11,329 (13.3) | 0.02 |
| 2008 | 81,675 (12.5) | 12,249 (14.3) | 0.05 |
| 2009 | 81,217 (12.4) | 12,766 (14.9) | 0.07 |
| 2010 | 79,396 (12.1) | 14,727 (17.2) | 0.14 |
| 2011 | 76,912 (11.8) | 4,070 (4.8) | 0.26 |
| 2012 | 206 (0.0) | 0 (0.0) | 0.31 |
| ***Maternal factors*** |  |  |  |
| Maternal age at child’s birth (years) Mean (SD) | 30.2 (5.6) | 29.3 (5.9) | 0.15 |
| <25 | 105,537 (16.1) | 20,334 (23.8) | 0.19 |
| 25 to 34 | 398,680 (61.0) | 47,109 (55.1) | 0.12 |
| 35+ | 149,443 (22.9) | 18,035 (21.1) | 0.04 |
| Born in a Western Country | 142,306 (21.8) | 17,164 (20.1) | 0.04 |
| Does not have a partner | 83,194 (12.7) | 30,570 (35.8) | 0.56 |
| Social security benefits received^a^ | 55,828 (8.5) | 19,120 (22.4) | 0.39 |
| Parity |  |  |  |
| First born child | 309,383 (47.3) | 6,649 (7.8) | 0.99 |
| Second born child | 218,512 (33.4) | 28,763 (33.6) | 0.01 |
| Third born child | 85,933 (13.1) | 24,271 (28.4) | 0.38 |
| Fourth born child or more | 39,832 (6.1) | 25,795 (30.2) | 0.66 |
| Previous caesarean delivery | 84,108 (12.9) | 13,862 (16.2) | 0.10 |
| Smoking during pregnancy | 12,939 (2.0) | 7,600 (8.9) | 0.31 |
| Quintiles of area-based socioeconomic disadvantage (IRSD) |  |  |  |
| Most disadvantaged | 138,674 (21.2) | 38,009 (44.5) | 0.51 |
| 2 | 112,947 (17.3) | 20,474 (24.0) | 0.17 |
| 3 | 134,627 (20.6) | 15,582 (18.2) | 0.06 |
| 4 | 99,119 (15.2) | 6,425 (7.5) | 0.24 |
| Least disadvantaged | 168,293 (25.7) | 4,988 (5.8) | 0.57 |
| Area-based residential remoteness (ARIA) |  |  |  |
| Major Cities of Australia | 520,378 (79.6) | 56,852 (66.5) | 0.30 |
| Inner Regional Australia | 98,935 (15.1) | 19,921 (23.3) | 0.21 |
| Outer Regional Australia | 31,063 (4.8) | 7,864 (9.2) | 0.18 |
| Remote & Very Remote Australia | 3,284 (0.5) | 841 (1.0) | 0.06 |
| Multifetal gestation | 19,160 (2.9) | 2,518 (2.9) | 0.00 |
| ***Maternal conditions (year prior to LMP until the day before childbirth)*** | | | |
| Cardiovascular disease | 3,322 (0.5) | 2,028 (2.4) | 0.16 |
| Cancer diagnosis or treatment | 65 (0.0) | 7 (0.0) | 0.00 |
| Chronic liver disease | 171 (0.0) | 35 (0.0) | 0.01 |
| Chronic renal disease | 230 (0.0) | 60 (0.1) | 0.02 |
| Drug and alcohol disorder | 2,538 (0.4) | 944 (1.1) | 0.08 |
| Epilepsy | 297 (0.0) | 101 (0.1) | 0.03 |
| Pre-existing hypertension | 2,496 (0.4) | 419 (0.5) | 0.02 |
| Pre-existing diabetes | 4,116 (0.6) | 391 (0.5) | 0.02 |
| Mental health | 34,615 (5.3) | 13,539 (15.8) | 0.35 |
| Severe mental health | 2,505 (0.4) | 1,234 (1.4) | 0.11 |
| Obesity | 511 (0.1) | 185 (0.2) | 0.04 |
| Respiratory disease | 18 (0.0) | 12 (0.0) | 0.01 |
| Thyroid disease | 3,424 (0.5) | 1,382 (1.6) | 0.11 |
| Chronic viral hepatitis and HIV | 1,029 (0.2) | 308 (0.4) | 0.04 |
| Rheumatic disease | 2,934 (0.4) | 336 (0.4) | 0.01 |
| Surgery^b^ | 46 (0.0) | 9 (0.0) | 0.00 |
| Musculoskeletal pain^b^ | 111 (0.0) | 36 (0.0) | 0.02 |
| ***Pre-pregnancy maternal healthcare utilisation (year prior to LMP)*** | | | |
| Number of hospital admissions |  |  |  |
| 0 | 482,498 (73.8) | 59,442 (69.5) | 0.10 |
| 1 | 122,686 (18.8) | 18,657 (21.8) | 0.08 |
| ≥2 | 48,476 (7.4) | 7,379 (8.6) | 0.05 |
| Number of ED presentations |  |  |  |
| 0 | 574,538 (87.9) | 68,132 (79.7) | 0.22 |
| 1 | 53,751 (8.2) | 10,373 (12.1) | 0.13 |
| ≥2 | 25,371 (3.9) | 6,973 (8.2) | 0.18 |
| Number of GP visits |  |  |  |
| 0 | 361,926 (55.4) | 43,030 (50.3) | 0.10 |
| 1 | 159,646 (24.4) | 21,306 (24.9) | 0.01 |
| ≥2 | 132,088 (20.2) | 21,142 (24.7) | 0.11 |

Abbreviations: ED, Emergency Department; GP, General practitioners; HIV, Human Immunodeficiency Virus; SD, Standard Deviation.
^a^ Social security benefits refer to government-administered financial assistance provided to eligible individuals.
^b^ Ascertained in the year prior to last menstrual period until the last menstrual period. .

Table S9: STROBE Statement—Checklist of items that should be included in reports of *cohort studies*

|  | Item No | Recommendation | Page No |
| --- | --- | --- | --- |
| **Title and abstract** | 1 | (*a*) Indicate the study’s design with a commonly used term in the title or the abstract | 1, 5 |
|  |  | (*b*) Provide in the abstract an informative and balanced summary of what was done and what was found | 5, 6 |
| Introduction | | | |
| Background/rationale | 2 | Explain the scientific background and rationale for the investigation being reported | 7 |
| Objectives | 3 | State specific objectives, including any prespecified hypotheses | 7 |
| Methods | | | |
| Study design | 4 | Present key elements of study design early in the paper | 7, 8 |
| Setting | 5 | Describe the setting, locations, and relevant dates, including periods of recruitment, exposure, follow-up, and data collection | 7, 8, eAppendix 1, Figure S1 |
| Participants | 6 | (*a*) Give the eligibility criteria, and the sources and methods of selection of participants. Describe methods of follow-up | 8 |
|  |  | (*b*) For matched studies, give matching criteria and number of exposed and unexposed | NA |
| Variables | 7 | Clearly define all outcomes, exposures, predictors, potential confounders, and effect modifiers. Give diagnostic criteria, if applicable | 8, 9, 10, Table S2, Table S3 |
| Data sources/ measurement | 8* | For each variable of interest, give sources of data and details of methods of assessment (measurement). Describe comparability of assessment methods if there is more than one group | Appendix 1, Table S3 |
| Bias | 9 | Describe any efforts to address potential sources of bias | 10, 11, 12 |
| Study size | 10 | Explain how the study size was arrived at | 8, 9, 10, Figure 1 |
| Quantitative variables | 11 | Explain how quantitative variables were handled in the analyses. If applicable, describe which groupings were chosen and why | 9, 10 |
| Statistical methods | 12 | (*a*) Describe all statistical methods, including those used to control for confounding | 10 |
|  |  | (*b*) Describe any methods used to examine subgroups and interactions | 10, 11 |
|  |  | (*c*) Explain how missing data were addressed | 10 |
|  |  | (*d*) If applicable, explain how loss to follow-up was addressed | 10, 11 |
|  |  | (*e*) Describe any sensitivity analyses | 11, 12 |
| Results | | |  |
| Participants | 13* | (a) Report numbers of individuals at each stage of study—eg numbers potentially eligible, examined for eligibility, confirmed eligible, included in the study, completing follow-up, and analysed | 12, Figure 1 |
|  |  | (b) Give reasons for non-participation at each stage | 12, Figure 1 |
|  |  | (c) Consider use of a flow diagram | Figure 1 |
| Descriptive data | 14* | (a) Give characteristics of study participants (eg demographic, clinical, social) and information on exposures and potential confounders | 12, 13, Table 1 |
|  |  | (b) Indicate number of participants with missing data for each variable of interest | Table S3 |
|  |  | (c) Summarise follow-up time (eg, average and total amount) | Table S6 |
| Outcome data | 15* | Report numbers of outcome events or summary measures over time | Figure 2, 3, Figure S6, S7 |

| Main results | 16 | (*a*) Give unadjusted estimates and, if applicable, confounder-adjusted estimates and their precision (eg, 95% confidence interval). Make clear which confounders were adjusted for and why they were included | 13 |
| --- | --- | --- | --- |
|  |  | (*b*) Report category boundaries when continuous variables were categorized | NA |
|  |  | (*c*) If relevant, consider translating estimates of relative risk into absolute risk for a meaningful time period | NA |
| Other analyses | 17 | Report other analyses done—eg analyses of subgroups and interactions, and sensitivity analyses | 13, 14 |
| Discussion | | | |
| Key results | 18 | Summarise key results with reference to study objectives | 14 |
| Limitations | 19 | Discuss limitations of the study, taking into account sources of potential bias or imprecision. Discuss both direction and magnitude of any potential bias | 15, 16 |
| Interpretation | 20 | Give a cautious overall interpretation of results considering objectives, limitations, multiplicity of analyses, results from similar studies, and other relevant evidence | 15, 16 |
| Generalisability | 21 | Discuss the generalisability (external validity) of the study results | 16 |
| Other information | | | |
| Funding | 22 | Give the source of funding and the role of the funders for the present study and, if applicable, for the original study on which the present article is based | 2 |

**References:**

1. Department of Health and Ageing Trends in and drivers of Pharmaceutical Benefits Scheme expenditure 2013, (Canberra, Australia, 2023, accessed November 28, 2024), <http://wwwpbsgovau/statistics/asm/2010/australian-statistics-on-medicine-2010pdf>. .

2. Australian Commission on Safety and Quality in Health Care. Medication Safety Standard. Available from: <https://www.safetyandquality.gov.au/standards/nsqhs-standards/medication-safety-standard> (Accessed: 04 June 2024).

3. Sun JW, Bourgeois FT, Haneuse S, Hernandez-Diaz S, Landon JE, Bateman BT, et al. Development and Validation of a Pediatric Comorbidity Index. Am J Epidemiol. 2021;190(5):918-27.

4. Joseph FA, Hyett JA, Schluter PJ, McLennan A, Gordon A, Chambers GM, et al. New Australian birthweight centiles. Med J Aust. 2020;213(2):79-85.

5. Raichand S, Pearson SA, Zoega H, Buckley NA, Havard A. Utilisation of teratogenic medicines before and during pregnancy in Australian women. Aust N Z J Obstet Gynaecol. 2020;60(2):218-24.

6. Lain SJ, Roberts CL, Hadfield RM, Bell JC, Morris JM. How accurate is the reporting of obstetric haemorrhage in hospital discharge data? A validation study. Aust N Z J Obstet Gynaecol. 2008;48(5):481-4.

hospital discharge data for determining neonatal morbidity and mortality: a validation study. BMC Health Services Research. 2007;7(1):188.
